# Metal single-site catalyst design for electrocatalytic production of hydrogen peroxide at industrial-relevant currents

**DOI:** 10.1038/s41467-023-35839-z

**Published:** 2023-01-12

**Authors:** Peike Cao, Xie Quan, Xiaowa Nie, Kun Zhao, Yanming Liu, Shuo Chen, Hongtao Yu, Jingguang G. Chen

**Affiliations:** 1 Key Laboratory of Industrial Ecology and Environmental Engineering (Ministry of Education, China), School of Environmental Science and Technology, Dalian University of Technology, Dalian, 116024 PR China; 2 State Key Laboratory of Fine Chemicals, Frontiers Science Center for Smart Materials, School of Chemical Engineering, Dalian University of Technology, Dalian, 116024 PR China; 3 College of Environmental Science and Engineering, North China Electric Power University, Beijing, 102206 PR China; 4 Department of Chemical Engineering, Columbia University, New York, NY 10027 USA

**Keywords:** Pollution remediation, Electrocatalysis

## Abstract

Direct hydrogen peroxide (H_2_O_2_) electrosynthesis via the two-electron oxygen reduction reaction is a sustainable alternative to the traditional energy-intensive anthraquinone technology. However, high-performance and scalable electrocatalysts with industrial-relevant production rates remain to be challenging, partially due to insufficient atomic level understanding in catalyst design. Here we utilize theoretical approaches to identify transition-metal single-site catalysts for two-electron oxygen reduction using the *OOH binding energy as a descriptor. The theoretical predictions are then used as guidance to synthesize the desired cobalt single-site catalyst with a O-modified Co-(pyrrolic N)_4_ configuration that can achieve industrial-relevant current densities up to 300 mA cm^−^^2^ with 96–100% Faradaic efficiencies for H_2_O_2_ production at a record rate of 11,527 mmol h^−^^1^ g_cat_^−^^1^. Here, we show the feasibility and versatility of metal single-site catalyst design using various commercial carbon and cobalt phthalocyanine as starting materials and the high applicability for H_2_O_2_ electrosynthesis in acidic, neutral and alkaline electrolytes.

## Abstract (web summary)

Direct hydrogen peroxide electrosynthesis offers a sustainable alternative to the traditional energy-intensive anthraquinone technology. Here, the authors report a scalable cobalt single-site catalyst for hydrogen peroxide synthesis at industrial-relevant currents in acidic, neutral or alkaline electrolyte.

## Introduction

Hydrogen peroxide (H_2_O_2_) has been widely used in industrial processes including in fiber and paper production, water treatment and environmental remediation in acidic and neutral mediums^1–4^. Currently, commercial H_2_O_2_ manufacture primarily relies on the large-scale anthraquinone oxidation process; however, this technology requires enormous capital input for infrastructure, and is energy-intensive accompanied by substantial quantities of hazardous waste discharge during operation. In order to minimize the transportation cost from manufacturing point to end-users, H_2_O_2_ is concentrated up to 70 wt%, which poses safety issues due to potential explosion risk and brings about extra carbon footprint. Additionally, the addition of stabilizers to prevent H_2_O_2_ degradation often negatively affects the chemical activity of H_2_O_2_, while the chemical residues also create concerns in water treatment applications. Thus, it is highly desired to realize sustainable production of H_2_O_2_ using electric energy that would enable portable and safe on-demand H_2_O_2_ supply for decentralized or remote locations.

Recent advances demonstrate that the electrocatalytic two-electron oxygen reduction reaction (ORR) with H_2_O and O_2_ as reactants provides a promising route for direct H_2_O_2_ production^1,5–8^. Carbonaceous catalysts show high two-electron ORR selectivity but a low intrinsic activity compared to metal-based catalysts due to weak *OOH binding^9,10^. Platinum-group metals are recognized as the most active ORR catalysts^11–13^, but for exclusive four-electron pathway to H_2_O due to the strong *OOH binding on adjacent metal atoms to cause O–O breakage. Theoretically, the activity and selectivity of the two-electron ORR follow the Sabatier volcano-type dependence on the *OOH binding, where the optimal performance can be achieved with a suitable *OOH binding energy that situates at the volcano peak^11,14,15^. Stephens and Rossmeisl, et al. proposed the spatial site isolation strategy of reactive metal atoms using inert Au or Hg elements, and discovered the PtHg_4_ alloy with outstanding electrocatalytic property for H_2_O_2_ production by computational screening using the density functional theory (DFT) method^14^. Chorkendorff and Stephens, et al. identified Ag−Hg and Pd−Hg catalysts highly active and selective for H_2_O_2_ production by searching the optimal *OOH binding closest to the volcano peak^15^. Therefore, the theoretically calculated two-electron ORR volcano plot with the *OOH binding as the descriptor can be regarded as one effective tool to discover new catalysts for H_2_O_2_ electrosynthesis by computational screening.

Metal single-site catalysts (SSCs) have the isolated and active sites with the adjustable electronic property by regulating the metal centers and the coordination configurations, which provides the opportunities for the *OOH binding tuning^5,6,16^. Recent studies indicate that the transition-metal SSCs, such as Co, Fe and Ni, show the electrocatalytic activities for H_2_O_2_ production^3,17–20^. For example, iron single atoms coordinated with C and O atoms exhibited high H_2_O_2_ selectivity, in contrast to the well-known FeN_4_ for H_2_O formation^3,21^. The Co SSCs have attracted extensive attentions due to the more suitable *OOH binding for H_2_O_2_ production, however, the widely known CoN_4_ are less selective for H_2_O_2_ due to the strong *OOH binding^18^. It was reported that introducing the oxygen-containing groups around the CoN_4_ sites could weaken *OOH binding and enhance the H_2_O_2_ selectivity^17–19^. However, the electronic effects induced by configurational variation of the active metal moiety and the atomic level modification mechanisms still remain insufficiently understood for the two-electron ORR. Moreover, it is often difficult to achieve scalable synthesis of well-defined SSCs with atomic level control due to the inhomogeneity and the random dispersion of metal sites. The practical electrocatalytic H_2_O_2_ synthesis still faces great challenges of achieving both commercially relevant currents and H_2_O_2_ Faradaic efficiency (FE), which is often characterized by low H_2_O_2_ production rate and product concentration. Thus, it is critical to develop implementable design for developing high-performance and low-cost two-electron ORR catalysts for high-efficiency H_2_O_2_ electrosynthesis.

Here, we report oxygen groups modified transition-metal phthalocyanine (TMPc) catalysts with TM-(pyrrolic N)_4_ configuration for high-performance H_2_O_2_ synthesis in acidic, neutral and alkaline electrolytes. The electronic properties of TMPc are adjusted by changing the metal centers and the adjacent coordination atoms from oxidized carbon nanotube (OCNT) support, thus enhancing the H_2_O_2_ selectivity. DFT calculations are conducted to screen for highly selective and active two-electron ORR electrocatalysts among TMPc catalysts by establishing the activity-volcano plot with the *OOH binding energy as the descriptor. The optimal *OOH binding at the volcano peak is achieved for cobalt phthalocyanine molecule anchored on oxygen-modified carbon plane (CoPc-OCNT), which can be ascribed to the electronic state modification below the Fermi level. The prepared CoPc-OCNT catalyst shows the high activity and exclusive selectivity for H_2_O_2_ electrosynthesis in alkaline electrolyte, where the industrial-relevant current density up to 300 mA cm^−^^2^ is achieved at a remarkable production rate while maintaining high FE. The CoPc-OCNT catalyst also obtains good H_2_O_2_ electrosynthesis performance using a neutral or acidic electrolyte, which is applicable for wastewater treatment. It also demonstrates the versatility of designing oxygen groups modified Co SSCs for H_2_O_2_ electrosynthesis by employing a variety of commercial carbon materials, showing the promising potential in scalable catalyst preparation.

## Results

### Theoretical calculations

**Fig. 1:**
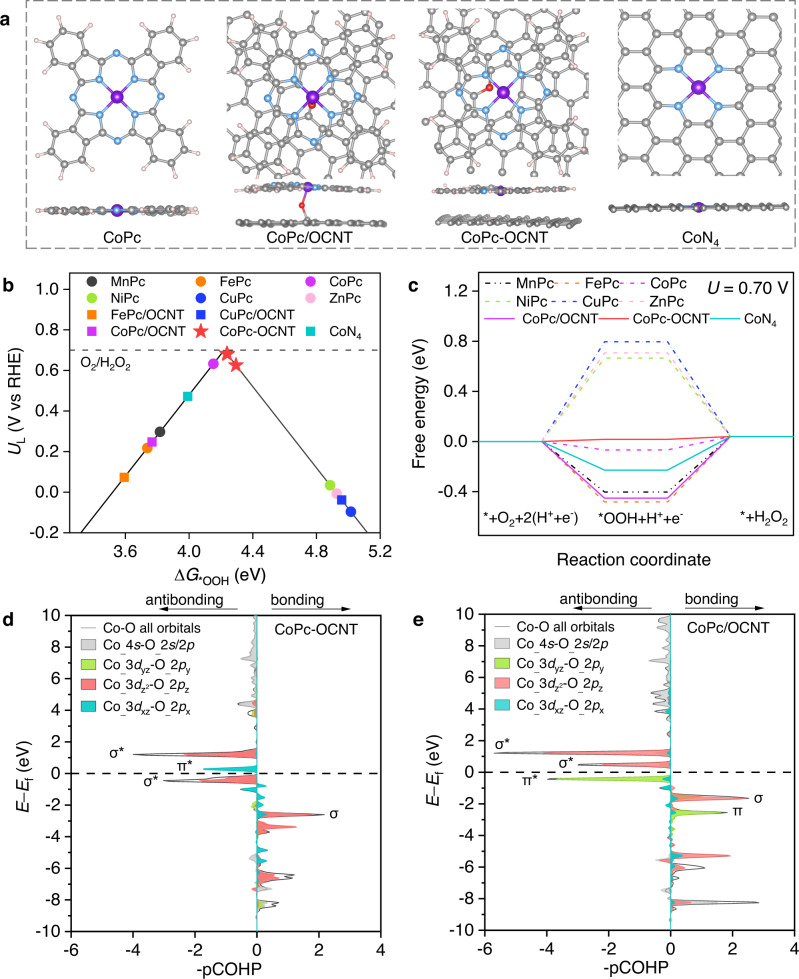
Theoretical calculations for two-electron ORR. **a** Atomic structures of CoPc, CoPc/OCNT, CoPc-OCNT and CoN_4_ models. **b** Calculated activity-volcano curve for two-electron ORR. **c** Free-energy diagrams for two-electron ORR pathway at 0.7 V vs RHE, where * and *OOH denote an unoccupied active site and adsorbed *OOH, respectively. **d–e** Projected crystal orbital Hamilton populations (pCOHP) between Co and p-orbital of O of *OOH on CoPc/OCNT and CoPc-OCNT.

DFT calculations are conducted to screen for highly active and selective two-electron ORR electrocatalyst among TMPc including MnPc, FePc, CoPc, NiPc, CuPc and ZnPc (Fig. 1a and Supplementary Figs. 1, 2). Figure 1b shows the volcano plot describing the two-electron ORR activity as a function of the Gibbs free energy for *OOH adsorption (ΔG_*OOH_). MnPc and FePc, situated on the left side of volcano plot, show strong *OOH binding to favorably dissociate the O–O bond adsorbed on active metal sites, resulting in selective H_2_O formation over H_2_O_2_^14,15^. NiPc, CuPc and ZnPc, located at the right side, present weak *OOH binding and thus high selectivity to H_2_O_2_ formation; however, the ORR activities are proposed to be inferior due to the rate-limiting protonation of O_2_ toward *OOH. In contrast, CoPc shows a ΔG_*OOH_ value of 4.15 eV that is closest to the activity-volcano peak (~4.22 eV)^14^, demonstrating the highest activity and selectivity toward the two-electron ORR among all TMPc assessed here. Free energy diagrams for the two-electron ORR in Fig. 1c display the largely uphill barrier, up to 0.66–0.80 eV, to form the *OOH intermediate on NiPc, CuPc and ZnPc at 0.7 V, confirming the poor ORR activities. In contrast, the energy barrier of 0.40–0.48 eV for *OOH dissociation on both MnPc and FePc, originated from the excessively downhill step to form the *OOH, would drastically reduce the selectivity to H_2_O_2_. In line with the activity-volcano relationship, CoPc shows the lowest energy barrier of 0.07 eV for the entire reaction process of oxygen reduction to H_2_O_2_ formation, although there is still a small energy gap to the ideal reaction process where the free energy change is flat with zero energy barrier^11,15^. It is worth noting that the ΔG_*OOH_ values of MnPc, FePc, CoPc, NiPc and CuPc show the correlation with the d-band center (Supplementary Figs. 3, 4), where the lower d-band center of metal atoms contributes to more positive ΔG_*OOH_ and thus weaker binding ability^22,23^. Furthermore, the adjustment of adjacent atomic environment of the metal sites can also effectively optimize the *OOH binding by modifying the electronic structures^17–19^. Compared to CoPc, the too strong *OOH binding away from the top of the volcano plot on the typical CoN_4_ structure suggests the possibility of tuning ORR selectivity by coordinating the Co center. The O-modified graphitic carbon is employed to anchor the CoPc molecule for modifying the local configuration of the Co center. It’s worth noting that O modified graphitic carbon have poor ORR activity due to weak *OOH binding, while the Co site of CoPc has good *OOH binding and is highly ORR active that can be ascribed to the high electrophilicity to *OOH (Supplementary Figs. 5–7). Interestingly, it is found that different types of O atoms on the carbon plane (Supplementary Fig. 8) result in two opposite effects that strengthen or weaken the *OOH binding on CoPc. The O dopant at the defective carbon site helps the Co site (denoted as CoPc-OCNT) achieve a slightly increased ΔG_*OOH_ that is closest to the activity-volcano peak (Fig. 1b). The corresponding free energy diagram exhibits a negligible uphill energy barrier (Fig. 1c), suggesting excellent two-electron ORR activity of CoPc-OCNT. However, axial O coordination at the carbon basal plane to the Co center (denoted as CoPc/OCNT) causes a stronger *OOH binding with a reduced ΔG_*OOH_ of 3.77 eV and increases the energy barrier (0.45 eV) for H_2_O_2_ formation, adversely reducing the two-electron selectivity. The effect of axial O coordination is also confirmed on FePc or CuPc, which causes stronger *OOH binding and thus lower H_2_O_2_ selectivity on Fe site while moderately improved ORR activity on Cu site (Supplementary Fig. 9). We further examine the H_2_O_2_ selectivity of catalysts by inspecting the entire ORR process containing three primitive steps of *+O_2_→*OOH→*H_2_O_2_→*+H_2_O_2_ as well as the O‒O bond dissociation of the adsorbed *OOH or *H_2_O_2_ (Supplementary Fig. 10). The CoPc-OCNT catalyst shows a small energy barrier during the entire two-electron ORR process, indicating a low overpotential for producing H_2_O_2_. It is important to note that the thermodynamically favorable O‒O bond breakage via the dissociation of *H_2_O_2_ or *OOH over H_2_O_2_ formation does not imply a low H_2_O_2_ selectivity as the kinetic energy barrier under real electrocatalytic conditions is likely high enough to prevent the O‒O from breaking^24,25^ (see more analysis in Supporting Information). To bridge the gap between the theoretical models and real catalytic configurations, several types of possible OCNT structures with O dopant locating at different defective sites are considered as the supports of CoPc (shown in Supplementary Fig. 11). They show similar ΔG_*OOH_ values, suggesting that the C‒O‒O group at the defective site can effectively modify the Co sites of CoPc to achieve an optimal *OOH binding energy. Furthermore, we find that cobalt tetrabenzoporphyrin (CoTBP) with four pyrrolic N coordination supported by OCNT also show the optimal *OOH binding energy that is close to the peak of the volcano (Supplementary Fig. 12). Thus, we infer that Co sites of these Co SSC coordinated with pyrrolic N are intrinsically active to selectively catalyze two-electron ORR.

To uncover the origin for ORR selectivity tuning on Co SSC, the electronic properties of the Co atom in CoPc/OCNT and CoPc-OCNT are analyzed by studying the density of states and Bader charge. Projected density of states (PDOS) of CoPc/OCNT and CoPc-OCNT in Supplementary Fig. 13 reveal that the different degree of orbital hybridization before and after *OOH adsorption determine the properties of *OOH adsorption. We calculate the projected crystal orbital Hamilton populations (pCOHP) between the Co atom and the O atom of *OOH to explain the bond strength (Fig. 1d). The pCOHP of CoPc/OCNT shows that all antibonding σ* orbitals are located at unoccupied states above the Fermi level, which contributes to reinforced *OOH adsorption. In contrast, pCOHP of CoPc-OCNT reveals that antibonding electrons of the σ* orbital with highly occupied states below the Fermi level reduce the strength of the Co‒O bond, which well accounts for weaker *OOH adsorption. Moreover, the absence of the back-donation effect due to antibonding π* with unoccupied state above the Fermi level is also responsible for the weak Co‒O bond in CoPc-OCNT. Bader charge analysis indicates charge loss at the Co site and charge accumulation at the O atom after *OOH is adsorbed (Supplementary Figs. 14,15 and Supplementary Table 1)), suggesting charge transfer from the Co center to O atoms. More charge lose on Co atom in CoPc-OCNT (−0.898 |*e*|) compared with CoPc (−0.898 |*e*|) and CoPc/OCNT (−0.612 |*e*|) suggests the intensified electron transfer from Co to surrounding ligands (Supplementary Figs. 16,17), which is responsible for the weaker *OOH binding.

### Metal single-site catalyst synthesis and characterization

**Fig. 2:**
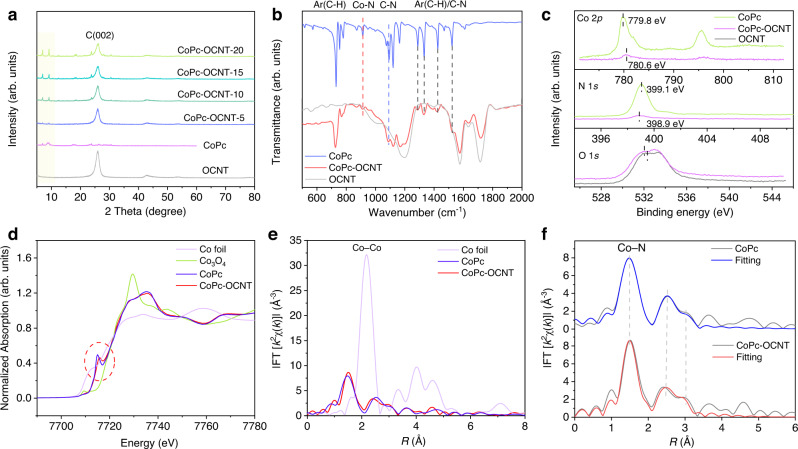
Characterizations of Co sites in the CoPc-OCNT catalyst. **a** XRD patterns of CoPc-OCNT with different Co loadings. **b** FT-IR spectra of OCNT, CoPc-OCNT and CoPc reference. **c** Co 2*p*, N 1*s* and O 1*s* high-resolution XPS spectra of CoPc-OCNT and the reference materials (Co foil, Co_3_O_4_ and CoPc). **d** XANES and **e–f** FT-EXAFS curves at *R* space of Co K-edge and the corresponding fittings for CoPc-OCNT and the reference materials (Co foil and CoPc).

Guided by the theoretical predictions, TMPc-OCNT (TM=Fe, Co or Cu) catalysts are synthesized by combining TMPc with the OCNT support through a sufficient mixing process (see Methods). This mild synthetic method at room temperature is adopted to avoid generating the strong bonding between the TM site in TMPc and the O atom in OCNT and to prevent the decomposition of TMPc molecules in typical high-temperature processing. The OCNT material is prepared by oxidatively treating the original CNT (ori-CNT) to create oxygen-containing functional groups. As illustrated in Supplementary Fig. 18, no visible morphology changes are observed on OCNT after oxidation. X-ray photoelectron spectroscopy (XPS) measurements show that OCNT has a significantly increased O content (9.1%) compared to ori-CNT (2.5%), and these introduced oxygen functional groups exist mainly in the form of C–O–C (O at defective site) as well as minor portion of C=O (O on carbon plane)^18,26^, as also revealed by Fourier transform infrared (FT-IR) spectra (Supplementary Fig. 19). Raman spectra reveal an obvious increasement of carbon defects in OCNT due to more O dopant. The analysis of N_2_ adsorption-desorption isotherms shows a two-fold increase in the specific surface area of OCNT compared to ori-CNT (Supplementary Fig. 20), which is favorable to provide more opportunities to anchor TMPc molecules. The atomic contents of Fe, Co and Cu are determined by XPS to be 0.57, 0.30 and 0.79 at% on the surface of FePc-OCNT, CoPc-OCNT and CuPc-OCNT, respectively, (Supplementary Fig. 21). The X-ray diffraction (XRD) patterns show that all TMPc-OCNT catalysts contain the characteristic diffraction peaks that are well-matched with reference TMPc molecules (Supplementary Fig. 22), suggesting that the molecular structures of TMPc are retained after being anchored on OCNT. The XRD patterns for CoPc-OCNT (Fig. 2a) show that the characteristic peaks located at 7.0° and 9.2° are markedly increased in intensity when the CoPc content is increased from 0.23 to 0.45 at% (Supplementary Tables 2, 3)^27^, indicating that the loading of molecular CoPc can be adjustable by increasing concentration. The FT-IR results of CoPc-OCNT display the characteristic vibrational peaks located at 435–873, 915 and 1090–1524 cm^−^^1^ that are assigned to the C–H/C–C bending, Co–N and C–H/C–N stretching (Fig. 2b and Supplementary Table 4), respectively, being in good agreement with the CoPc reference and confirming the undamaged molecular CoPc structure anchored on OCNT^27,28^. Similar results are also observed on CuPc-OCNT and FePc-OCNT (Supplementary Fig. 23), indicating the successful CuPc and FePc loading. Furthermore, we examine the chemical interactions between Co of CoPc and O of the OCNT by high-resolution XPS spectra, which reveals a slight peak shift in the Co peak of CoPc-OCNT to higher binding energy comparing to CoPc, which is companied by a reverse shift of the O peak (Fig. 2c). Similar shifts of spectral peaks are also observed on FePc-OCNT and CuPc-OCNT but not on Pc-OCNT (Supplementary Fig. 24), indicating more positive valence for the metal sites, likely due to charge transfer from the metal centers to surrounding coordinated atoms^29–32^. Additionally, the O 1*s* high-resolution spectrum reveals that the C–O–C group is dominant in CoPc-OCNT compared to C=O. To identify the structural configuration of metal sites in CoPc-OCNT, X-ray absorption near edge spectroscopy (XANES) and extended X-ray absorption fine structure (EXAFS) of the Co K-edge are analyzed. As illustrated in Fig. 2d, the near-edge absorption intensity of the Co K-edge for CoPc-OCNT is between that of Co foil and Co_2_O_3_, suggesting a positively charged Co atom^32,33^. The detection of the pre-edge peak at 7715 eV (a signature of the square-planar Co–N_4_ structure in CoPc) indicates that the configuration of Co–N_4_ remains intact on OCNT^33–35^. Referring to the Fourier transformed EXAFS (FT-EXAFS) for Co foil (Fig. 2e and Supplementary Fig. 25), the Co–Co scattering path (2.2 Å) is absent in CoPc-OCNT, indicating that Co exists as isolated single sites. In comparison with CoPc, the EXAFS fitting for CoPc-OCNT shows a main peak at 1.5 Å and the minor peaks at 2.5 and 3.0 Å that can be assigned to the first scattering path of Co–N and the second paths of Co–C–N and Co–N–C, respectively (Fig. 2f). The quantitative fitting of the EXAFS spectra reveals the first coordination shell of four Co–N bonds at the distance of 1.9 Å and the second shell of Co–N–C and Co–C–N at the distances of 3.3 and 3.0 Å (Supplementary Fig. 26 and Supplementary Table 5). Overall, the results above reveal the CoPc-like Co-(pyrrolic N)_4_ structure with O atom modification in CoPc-OCNT.

### Electrocatalytic two-electron ORR performance

**Fig. 3:**
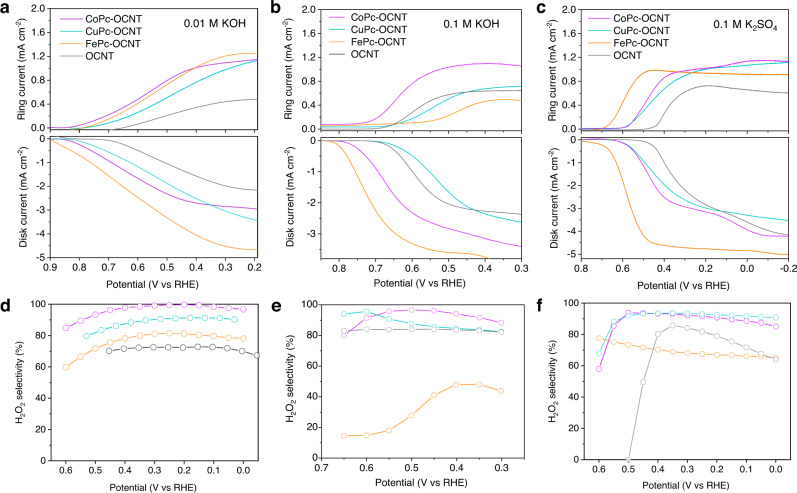
Two-electron ORR activity and selectivity evaluation of the TMPc-OCNT catalysts using RRDE. Linear sweep voltammetry show that ORR and simultaneous H_2_O_2_ oxidization currents are achieved on disk and ring electrodes, respectively. **a-b** Tests in 0.01 M KOH (pH 11.9) **c-d** 0.1 M KOH (pH 12.7) and **e-f** 0.1 M K_2_SO_4_ (pH 7.2).

The two-electron ORR activity and selectivity of TMPc-OCNT catalysts are investigated by rotating ring-disk electrode (RRDE) using a standard three-electrode system in 0.01 M KOH (pH 11.9), 0.1 M KOH (pH 12.7) and 0.1 M K_2_SO_4_ (pH 7.2) electrolytes. Catalyst is precisely casted on the disk region for catalyzing ORR to H_2_O_2_, and as-generated H_2_O_2_ is detected by the Pt ring electrode. The ORR and H_2_O_2_ oxidation currents in Fig. 3a exhibit that TMPc-OCNT catalysts show better activity with a more positive onset potential of 0.75–0.81 V vs RHE (defined at −0.1 mA cm^−^^2^ of H_2_O_2_ partial current)^36^ compared with OCNT (0.71 V vs RHE) in 0.01 M KOH (Supplementary Table 6). The negligible overpotentials on TMPc-OCNT for the two-electron ORR compared to the standard equilibrium potential of 0.76 V vs RHE in less alkaline solution suggests fast kinetics. Significantly, CoPc-OCNT exhibits the highest H_2_O_2_ selectivity of 95–100% in the potential range of 0–0.45 V vs RHE that is higher than that on CuPc-OCNT (86–91%), FePc-OCNT (75–81%) and OCNT (70–61%) (Fig. 3b). In 0.1 M KOH electrolyte, CoPc-OCNT also exhibits the highest H_2_O_2_ selectivity and activity among all tested catalysts (Fig. 3c, d). Noting that CuPc-OCNT shows a high H_2_O_2_ selectivity but lower activity, while FePc-OCNT has the opposite ORR performance, which is consistent with the theoretical predictions as aforementioned. Similar performance trends of catalysts are also observed in the neutral electrolyte of 0.1 M K_2_SO_4_ (Fig. 3e, f), and TMPc-OCNT catalysts show smaller overpotentials and higher H_2_O_2_ partial current densities compared with OCNT. Overall, CoPc-OCNT shows the best H_2_O_2_ activity and H_2_O_2_ partial current density in both alkaline and neutral conditions, being consistent with the theoretically predicted best performance from the volcano plot based on ΔG_*OOH_. The experimentally observed potential- or pH-dependent ORR activity and H_2_O_2_ selectivity are related with kinetics in electrocatalytic solid−liquid interfaces, which can be further explained by the simulation of kinetics involving water layers using advanced dynamic models^24,25^.

**Fig. 4:**
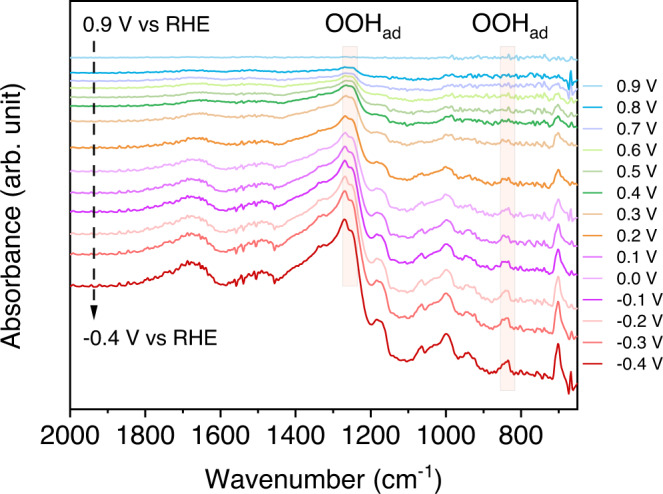
In situ detection of *OOH species. In situ ATR-SEIRAS spectra collected on CoPc-OCNT catalyst in O_2_-saturated 1.0 M KOH catholyte at different potentials from 0.9 to −0.4 V vs RHE (OOH_ad_: adsorbed *OOH).

In situ attenuated total reflectance surface-enhanced infrared absorption spectroscopy (ATR-SEIRAS) testes are performed to detect the key adsorbed OOH* on CoPc-OCNT during the electrolytic H_2_O_2_ synthesis (Supplementary Fig. 27). Figure 4 shows that a weak absorption band at about 1264 cm^−^^1^ appears when applying a potential of 0.8 V vs RHE, and this band is gradually enhanced by decreasing the potential. These absorption band on CoPc-OCNT can be assigned to O–O stretching vibration of *OOH, which are slightly shifted to higher wavenumber compared with the values in previous studies likely due to different adsorption sites^17,37,38^. Additionally, the bands at 835 cm^−^^1^ that increase with negatively shifted potential can be reasonably assigned to the M–O stretching mode of *OOH^39–41^. Moreover, the bands assigned to adsorbed hydroperoxide (typically at 1386 cm^−^^1^) are not detected^37^, because H_2_O^−^ product rather than H_2_O_2_ is produced at pH>11.6. Overall, the detection of the potential-dependent adsorbed hydroperoxy bands supports the *OOH mediated two-electron ORR pathway on the CoPc-OCNT catalyst.

**Fig. 5:**
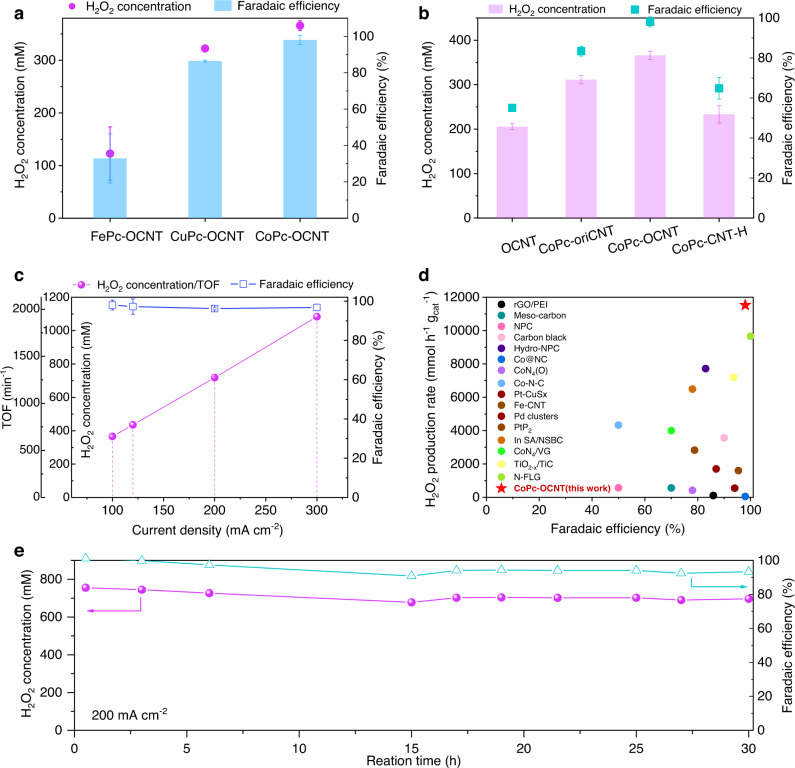
H_2_O_2_ electrosynthesis performance in a flow-cell electrolyzer based on the gas-diffusion cathode. **a, b** Concentrations of H_2_O_2_ directly outputted from electrolyzer and the Faradaic efficiency of FePc-OCNT, CoPc-OCNT, CuPc-OCNT, OCNT, CoPc-oriCNT or CoPc-CNT-H cathodes at 100 mA cm^−^^2^ (Data points and error bars represent the average and standard deviation of data from triplicate parallel tests). **c** H_2_O_2_ concentration, Faradaic efficiency and TOF values of CoPc-OCNT at 100, 120, 200 and 300 mA cm^−^^2^. **d** H_2_O_2_ production rate of CoPc-OCNT compared with most advanced catalysts newly reported (data sources are provided in Supplementary Table 8). **e** Stability evaluation of CoPc-OCNT cathode for continuous electrosynthesis of H_2_O_2_ at 200 mA cm^−^^2^ in a chronopotentiometry test. (Cathodic working area of 1 cm^−^^2^, the catholyte of 1.0 M KOH (pH 13.7) is one-pass flowed out at a rate of 5 mL h^−^^1^ to bring out the generated H_2_O_2_, while the anolyte of 0.5 M H_2_SO_4_ was circled at a rate of 33 mL h^−^^1^).

To evaluate the ability for electrocatalytically producing H_2_O_2_, the CoPc-OCNT catalyst is deposited on hydrophobic gas-diffusion electrode for enhancing O_2_ supply. We employ a two-electrode system assembled in a customized flow-cell reactor, as illustrated in Supplementary Fig. 28. As shown in Fig. 5a, the CoPc-OCNT can continuously produce 366 mM H_2_O_2_ solution with 98% FE at 100 mA cm^−^^2^, which is significantly greater than CuPc-OCNT (322 mM, 86%) and FePc-OCNT (123 mM, 33%). The H_2_O_2_ FE on both CoPc-OCNT and CuPc-OCNT are close to that measured by RRDE, suggesting high H_2_O_2_ selectivity and weak activity in decomposing H_2_O_2_. It is worth noting that unmodified OCNT shows moderate activity in producing H_2_O_2_ with 55% FE that is inferior to both CoPc-OCNT and CuPc-OCNT, indicating that metal sites in TMPc-OCNT are mainly responsible to the selectivity of H_2_O_2_ production. To demonstrate the effect of O coordination on the Co sites, O-containing groups on OCNT are removed by reductive thermal treatment to prepare CNT-H supported CoPc (CoPc-CNT-H). After the substantial elimination of O-containing groups from OCNT (confirmed by XPS in Supplementary Fig. 29), only 0.17% Co is loaded on the CNT-H support using the similar preparation method, which is lower than that on CoPc-OCNT (0.30%) and CoPc-oriCNT (0.48%, prepared using original CNT). Consequently, the CoPc-CNT-H exhibits poor H_2_O_2_ electrosynthesis performance (65% FE at 100–170 mA cm^−^^2^) than CoPc-OCNT and CoPc-oriCNT (96–98% and 67–83% FE at 100–200 mA cm^−^^2^) (Supplementary Fig. 30). Although a higher Co loading, CoPc-oriCNT deliveries more inferior Faradaic efficiency for producing H_2_O_2_ especially at larger currents compared to the CoPc-OCNT. It is inferred that there are moderately agglomerated CoPc molecules in the CoPc-oriCNT due to less strong interaction between Co sites and insufficient O-containing groups on oriCNT (Supplementary Fig. 29c), which demonstrates the crucial contribution of sufficient O modification to catalytic Co centers to the two-electron ORR selectivity.

Figure 5c shows that when increasing the current on the CoPc-OCNT cathode, the concentration of directly outflowed H_2_O_2_ almost linearly increases from 366 to 1084 mM (3.7 wt%) that can meet the concentration requirement for disinfection or wastewater treatment applications^42^ (3 wt% is generally sufficient). The CoPc-OCNT cathode can delivery ORR current density up to 300 mA cm^−^^2^ with remarkable FE of 96–100% that is higher than that achieved by majority of electrocatalysts currently reported and is comparable to the best values reported by Wang et al.^36,43^ (Supplementary Table 7 and Supplementary Fig. 31). To better understand the intrinsic activity, the turnover frequency (TOF) per metal site for H_2_O_2_ production is calculated. The CoPc-OCNT catalyst presents current-dependent TOF values that reach up to 649–1921 min^−^^1^ at 100–300 mA cm^−^^2^, demonstrating superior activity of the Co sites to Cu or Fe sites (Supplementary Figs. 32, 33). Significantly, the heterogeneous H_2_O_2_ electrosynthesis method shows great potential in maximizing the intrinsic activity of catalysts compared to homogeneous ORR that relies on Co-based molecular catalysts in organic system (limited TOF even at large overpotentials)^44^. The results from the current study show continuous H_2_O_2_ production in high-concentration, while which remains a great challenge for homogeneous molecular catalysis process that also suffers from H_2_O_2_ separation from the catalyst. Additionally, the CoPc-OCNT delivers a remarkable H_2_O_2_ production rate of 3892–11,527 mmol h^−^^1^ g_cat_^−^^1^ under the current densities of 100–300 mA cm^−^^2^ (Supplementary Fig. 34), higher than the values reported to the best of our knowledge (Fig. 5d and Supplementary Table 8). As shown in Fig. 5e, the continuous 30 h electrosynthesis of H_2_O_2_ at 200 mA cm^−^^2^ stably produces H_2_O_2_ at a concentration of 677–755 mM at a remarkable FE of 91–100%, demonstrating the electrochemical stability of the CoPc-OCNT electrode. In contrast, the activity and stability of CuPc-OCNT declines rapidly with increasing current, which might be ascribed to less robust bonding of CuPc on OCNT (Supplementary Fig. 32).

**Fig. 6:**
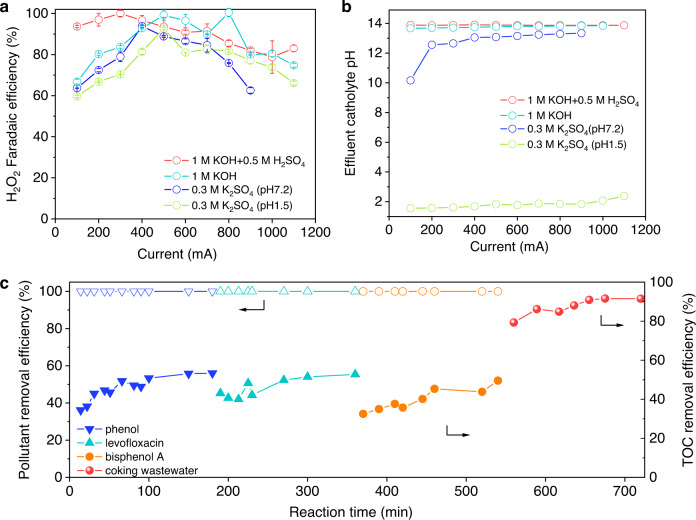
Acidic electrosynthesis H_2_O_2_ performance and Fenton applications. **a, b** H_2_O_2_ electrosynthesis employing 1 M KOH+0.5 M H_2_SO_4_, 1 M KOH+1 M KOH, 0.3 M K_2_SO_4_+0.3 M K_2_SO_4_ (pH 7.2 or 1.5) as catholyte and anolyte in the bigger flow-cell electrolyzer (test conditions: cathodic working area of 4 cm^−^^2^, catholyte and anolyte flow rates of 82–100 mL h^−^^1^). **c** Pollutant removal and TOC removal efficiency by the flow-cell Fenton system. (Test conditions: 500 mA current, effluent H_2_O_2_ rate of 72 mL h^−^^1^ synthetic wastewater: 10 mg  L^−^^1^ phenol, bisphenol A or levofloxacin and 1 mM Fe^2+^, flow rate of 700 mL h^−^^1^ real coking wastewater: initial TOC of 44.1 mg L^−^^1^, 5 mM Fe^2+^ dosage, flow rate of 140 mL h^−^^1^.).

We investigate the H_2_O_2_ electrosynthesis performance of CoPc-OCNT catalyst in less alkaline electrolyte (0.01 M KOH), which shows 97% H_2_O_2_ FE at 10 mA cm^−^^2^, but the FE drops to 27% at 20 mA cm^−^^2^ due to large overpotential (Supplementary Fig. 35a). The cell voltage can be significantly reduced by increasing electrolyte concentration or shortening the electrode distance to reduce solution resistance (Supplementary Figs. 35, 36). Results show that the cell voltage can be markedly reduced even in 0.01 M KOH, and the current density for H_2_O_2_ production is obviously lifted (Supplementary Fig. 35). When using 1 M KOH+0.5 M H_2_SO_4_ or 1 M KOH as catholyte and anolyte, the H_2_O_2_ FE are 83% and 75% at 1100 mA, and the partial H_2_O_2_ currents can reach 913 and 823 mA, respectively (Fig. 6a). The electrosynthesis process consumes an electric energy of 0.20–0.34 kWh for producing per kg 3 wt% H_2_O_2_ in the small reactor (Supplementary Fig. 37, calculations are described in Method) that is reduced to 0.12–0.28 kWh by the upgrading of the electrolyzer configuration, showing high-efficiency electrical energy conversion into valuable chemicals. Furthermore, the feasibility studies of scalable catalytic material preparation are explored, based on the simplicity of the CoPc-OCNT catalyst synthesis compared to the catalysts typically prepared by high-temperature carbonization. Five types of highly conductive, commercially available carbon materials are adopted as the supports for CoPc after introducing O-containing groups via the similar oxidization treatment to that of OCNT (Supplementary Figs. 38, 39). CoPc-OVX (Cabot VXC72) and CoPc-OAB (acetylene black) catalysts show higher FE of 85–98% for H_2_O_2_ production at 100–170 mA cm^−^^2^ compared to CoPc-OBP (black pearl 2000), CoPc-OECP (Ketjenblack ECP-600JD) and CoPc-OYP (Kurary YP-80F) (Supplementary Fig. 40). CoPc-OVX and CoPc-OAB can produce about 330 and 550 mM H_2_O_2_ at 100 and 150 mA cm^−^^2^ that are higher than that on CoPc-OBP, CoPc-OECP and CoPc-OYP. The performance of H_2_O_2_ electrosynthesis is closely related to the Co loading, and the best activity is achieved at a moderate loading while insufficient or excessive Co loading would degrade the activity and selectivity due to limited catalytic sites or CoPc aggregation. It’s worth noting that 0.2‒0.6 at% CoPc loadings on OCNT or commercial carbon materials approach the theoretical upper limit value of 0.6 at% that is estimated based on the densest monodisperse packing of CoPc on OCNT (described in Supplementary Fig. 41), where the space of adjacent Co sites is far enough to prevent the CoPc from aggregating and thus favor the two-electron ORR over the four-electron process. The results not only prove the wide versatility of the design of metal single-site catalysts, but also demonstrate the feasibility for scalable catalyst manufacture that is not limited by specific starting carbon materials. Furthermore, it is promising to prepare the high-density O-modified Co-(pyrrolic N)_4_ sites to improve the activity for H_2_O_2_ production by using the CoPc and commercial carbon materials as raw materials in further studies^24,25^.

The electrosynthesis of H_2_O_2_ is also explored in different pH for satisfying specific on-site applications related with environmental remediation that requires for acidic H_2_O_2_, such as Fenton reaction only effectively works pH ~3. When using 0.1 M K_2_SO_4_ catholyte and 0.5 M H_2_SO_4_ anolyte, about 70 mM acidic H_2_O_2_ can be produced at 20 mA cm^−2^ with 92% FE (Supplementary Fig. 42). To avoid additional potential differences brought by different electrolytes, we use a neutral or acidic electrolyte as both catholyte and anolyte to evaluate the performance of H_2_O_2_ electrosynthesis on CoPc-OCNT. In the bigger reactor, it can achieve 461–748 mA partial H_2_O_2_ currents with 75–92% FE at pH 1.5 and 375–607 mA partial H_2_O_2_ currents with 76–94% FE at pH 7.2 (Fig. 6a), which satisfy the concentration requirement from homogeneous or heterogeneous Fenton technology for wastewater treatment^45,46^. Notably, the CoPc-OCNT electrode shows exceptional high partial H_2_O_2_ currents in acidic electrolytes with the addition of 0.3 M K^+^, while majority of carbon-based catalysts show low FE for H_2_O_2_ production at current density over 50 mA cm^−^^2^. The performance enhancement is likely related to the effects of metal cations and drastic increase in local pH on electrode interface. The addition of metal cations was demonstrated to prevent further reduction of generated H_2_O_2_ to H_2_O by squeezing away the protons, thus promoting H_2_O_2_ selectivity^47^. The drastic increase in local pH on electrode–electrolyte interfaces due to the depletion of H^+^, especially when applying a current density over 100 mA cm^−^^2^, should also contribute to the enhanced H_2_O_2_ current density^48^. The experimentally observed pH increasements of effluent catholyte during electrosynthesis of H_2_O_2_ also confirm an increase in the pH (Fig. 6b). We highlight that such electrocatalytic reaction interfaces including metal cations and pH gradients are difficult to be accurately modelled by the DFT approach in this study based on purely thermodynamic analysis. Advanced computational methods, such as ab initio molecular dynamics simulations, are the potential tools to better describe and simulate the electrocatalytic properties of catalytic materials under real experimental conditions by considering the effects of solvent and ions and the kinetic information after a preliminary screening on the two-electron ORR catalysts^24,25,47,49^.

We built a flow-cell system combining electrosynthesis of H_2_O_2_ and the Fenton reaction for wastewater treatment, which shows almost 100% removal of the biodegradable pollutants with a treatment capacity of 700 mL h^−^^1^, wherein 40–52% total organic carbons (TOC) can be converted into CO_2_ (Fig. 6c). This flow-cell Fenton system also shows good performance for real coking wastewater treatment with almost 90% TOC removal. The 12 h acidic H_2_O_2_ electrosynthesis at 500 mA for refractory organic pollutant degradation and wastewater treatment by cooperating with the Fenton reaction demonstrates the potential in actual small-scale applications.

## Discussion

In summary, the ORR volcano plot with ΔG_*OOH_ as a key descriptor is established by DFT calculations to screen for highly selective two-electron electrocatalyst among TMPc catalysts. The ΔG_*OOH_ values of TMPc are associated with the d-band center of TM, which accounts for the ΔG_*OOH_ on CoPc being much closer to the peak of the volcano. The O dopant at the defective carbon site is demonstrated to modify the local electronic structure of the Co center in CoPc and helps achieve the ΔG_*OOH_ closest to the peak of the volcano. The prepared CoPc-OCNT catalyst with the O-modified Co-(pyrrolic N)_4_ configuration delivers high activity and H_2_O_2_ selectivity in both alkaline and neutral electrolytes, verifying the theoretically predicted activity-volcano trend for the two-electron ORR. The industrial-relevant current densities up to 300 mA cm^−^^2^ with 96–100% FE are achieved for continuous H_2_O_2_ production at a record rate of 11,527 mmol h^−^^1^ g_cat_^−^^1^ in a flow-cell electrolyzer. The high H_2_O_2_ production capacity at industrial-relevant currents is meaningful to help reduce infrastructure cost for promising scalable applications of H_2_O_2_ electrosynthesis. Furthermore, this study demonstrates the versatility of the metal single-site catalyst design using various commercial carbons as starting materials and high applicability for H_2_O_2_ electrosynthesis, presenting a promising potential in future large-scale H_2_O_2_ manufacture and in-situ wastewater treatment and disinfection.

## Methods

### Computational details

Density functional theory calculations were conducted using the Vienna Ab-initio Simulation Package (VASP 5.4.4 version)^50^. The electron-ion interactions and electron exchange-correlation interactions are described by the projector augmented wave (PAW) potential and Perdew-Burke-Ernzerhof (PBE) functional with the generalized gradient approximation (GGA) method^51,52^. The structural models of TMPc, OCNT and TMPc-OCNT were constructed in a cell with lattice parameters of 17 Å × 17 Å × 20 Å (Supplementary Figs. 1, 2 and 5). The *k*-point meshes of (1 × 1 × 1) was set. The spin polarization effect was considered for all calculations expect for OCNT. The cutoff energy of plane wave was set at 420 eV. All structural configurations were optimized until the forces on each atom were converged to less than 1 × 10^−^^2^ eV Å^−^^1^. Total free energy changes between two steps were less than 1 × 10^−^^5^ eV atom^−^^1^ in electronic relaxation. The two-electron ORR pathway is described as the following steps:

1 $$\ast+{{{{{{\rm{O}}}}}}}_{2}(g)+{{{{{{\rm{H}}}}}}}^{+}+{e}^-\to*{{{{{\rm{OOH}}}}}}$$

2 $$\ast {{{{{\rm{OOH}}}}}}+{{{{{{\rm{H}}}}}}}^{+}+{e}^-\to \ast+{{{{{{\rm{H}}}}}}}_{2}{{{{{{\rm{O}}}}}}}_{2}$$

where * and *OOH denote an unoccupied active site and adsorbed *OOH intermediate, respectively. Gibbs free energy (Δ*G*) for each step at the given potential *U* was calculated by the following equation:^14,53^

3 $$\varDelta G=\varDelta E+\varDelta ZPE+\varDelta {U}_{0\to T}-T\times \varDelta S+eU$$

where Δ*G* represents the free energy that is equal to the calculated Δ*E* after being corrected by zero-point energies (Δ*ZPE*) and entropic contributions (Δ*U*_0→T_ – *T*×*ΔS*) of adsorbates at 298.15 K. Δ*E* is the electronic energy difference between reactants and products for each step. These correction items can be conveniently calculated using a VASPKIT code^54^. *U* is the electrode potential versus reversible hydrogen electrode (vs RHE). It is worth pointing out that *G*(H^+^) is calculated by the equal of *G*(H^+^)=0.5 *G*(H_2_) – *G*(*e*) (pH=0, *U*=0 V), and *G*(O_2_) is calculated by the equal of 2 *G*(H_2_) + *G*(O_2_) – 2 *G*(H_2_O) = −4.92 eV.

During the two-electron ORR process, the limiting step is determined by both *OOH formation (Eq. (1)) and *OOH removal (Eq. (2)) from the catalytic sites. The theoretical overpotential is demonstrated to be a function of the *OOH binding energy, thus the limiting potential can be expressed as:^11^

4 $${U}_{{{{{{\rm{L}}}}}}1}=- \varDelta {G}_{\ast {{{{{\rm{OOH}}}}}}}+4.92$$

5 $${U}_{{{{{{\rm{L}}}}}}2}=\varDelta {G}_{\ast {{{{{\rm{OOH}}}}}}}-3.52$$

### Chemicals and materials

Commercial CNT powder (L-MWNT-2040) was purchased from Shenzhen Nanotech Port Co., Ltd. (Shenzhen, China). Cobalt phthalocyanine (CoPc, >95%), copper phthalocyanine (CuPc, >90.0%), phthalocyanine iron(II) (FePc, >98.0%), phthalocyanine (Pc, >93.0%), Ce(SO_4_)_2_ (99.9%), KOH (>95%) and K_2_SO_4_ (99%) were bought from Aladdin Co., Ltd. (Shanghai, China). N,N-dimethylformamide (DMF) was purchased from Bodi Chemical Reagent Co., Ltd, China. Nafion solution (5 wt%) was purchased from Du Pont Co., Ltd. Isopropanol was purchased from Tianjin Fuyu Fine Chemicals Co., Ltd. (China). Teflon-treated carbon fiber paper (HCP120) served as the support of GDE was provided by Hesen Electrical Co., Ltd. (Shanghai, China).

### Catalyst preparation

The OCNT material was prepared by chemically creating oxygen functional groups on the surface of original CNT, which was fulfilled by the oxidization treatment using concentrated nitric acid. In a typical operation, 3 g CNT power was homogeneously dispersed in 68 wt% HNO_3_ (150 mL) under continuous magnetic stirring. The oxidization treatment was conducted at 140 °C for 12 h, followed by water washing and drying. It is important to note that the evaporation of concentrated HNO_3_ and the decomposition products should be carefully treated via ice water condenser. In this process, the possible metal impurities (such as Fe, Ni, Cu, Co) on the surface of CNT would be removed after intensive acid etching. CoPc-OCNT, CuPc-OCNT, FePc-OCNT and Pc-OCNT catalysts were prepared using a simple impregnation method at room temperature. Typically, CoPc, CuPc, FePc or Pc (20 mg) molecules were dissolved in strong polar solvent of DMF (100 mL), followed by dropwise adding into the OCNT/DMF mixture (200/250 mg/mL) under rigorous stirring. The assembly process of phthalocyanines molecules and the OCNT support was conducted for 20 h, followed by separation and freeze-drying treatments. By adjusting the mass percent ratio of CoPc and OCNT using 10, 20, 30 or 40 mg CoPc with fixed 200 mg OCNT, CoPc-OCNT-5, CoPc-OCNT-10, CoPc-OCNT-15, and CoPc-OCNT-20 were prepared. As a comparison, CNT-H was prepared by removing the oxygen groups on OCNT under the reductive H_2_ atmosphere at 800 °C for 2 h. A control sample of CoPc-CNT-H was synthesized using the CNT-H support at 10% mass percent ratio of CoPc and CNT-H. Similarly, CoPc-ori-CNT was prepared using the original CNT as support.

### Catalyst characterization

The morphology of catalysts was observed by SEM (Hitachi S-4800) and TEM (Thermo-Tecnai G2 F30 S-Twin). The oxygen functional groups on OCNT and chemical constitutes of TMPc-OCNT were identified by FT-IR (RRUKER, VERTEX 70) and XPS (Thermos K-Alpha+ instrument with Al Kα X-ray excitation source). The phase structure was measured by XRD (Smartlab 9 kW, Nippon Neoku Electric Co. Ltd., Japan) and Raman (laser confocal microscopy Raman with laser excitation at 532 nm). The surface area and pore characteristics were assessed using N_2_ adsorption-desorption measurements (Quantachrome, Autosorb-IQ-C). The metal loadings of TMPc-OCNT were detected by XPS and inductively coupled plasma-mass spectrometry (ICP-MS, PerkinElmer, Nex ION 300D). The XAS was collected on the beamline BL01C1 at National Synchrotron Radiation Research Center (NSRRC), and the radiation was monochromatized by a Si (111) double-crystal monochromator. Ceshigo Research Service (Chengdu, China) provided the technical support for the data analysis of XANES and EXAFS that were processed using the Athena software^55^.

### Electrochemical measurements

The ORR performance of TMPc-OCNT catalysts was studied by RRDE device (AFMSRCE type, Pine, Physicochemical Co. Ltd. (Hong Kong)). Firstly, 2 mg of catalyst powder was well-dispersed in the mixture that consists of 0.9 mL H_2_O, 0.1 mL isopropanol and 20 μL of Nafion (5 wt%) via ultrasonication for 30 min for achieving the catalyst ink. The Pt counter electrode and Ag/AgCl reference electrode were used coupled with RRDE to construct the three-electrode system. The potential was normalized by converting to RHE according to the equation of *E* vs RHE=*E* vs Ag/AgCl+0.2+0.0591×pH. The potential versus RHE was adopted unless otherwise specified. The polarization current on the disk electrode and the H_2_O_2_ oxidation current on Pt ring electrode (fixed potential at 1.35 V vs RHE) were collected in O_2_ and Ar-saturated 0.01 M KOH electrolyte (pH 11.9) and 0.1 M K_2_SO_4_ (pH 7.2). The scan rate was set at 10 mV s^−^^1^ and the rotating speed was set at 1600 rpm. The collection efficiency N of H_2_O_2_ of the Pt ring electrode was calibrated to be 39%, which is theoretically based on one-electron reversible redox conversion of ferrocyanide/ferricyanide species^56,57^. It should be mentioned that the actual ORR current (*I*_R_) and H_2_O_2_ oxidation current (*I*_R_) were obtained by subtracting the values obtained in Ar-saturated solution from that in O_2_ condition. Based on the corrected currents, the H_2_O_2_ selectivity is calculated by the Eq. (7) of

6 $${{{\mbox{H}}}}_{2}{{{\mbox{O}}}}_{2}(\%)=200\times \frac{{I}_{{{\mbox{R}}}}/{{\mbox{N}}}}{{I}_{{{\mbox{D}}}}+{I}_{{{\mbox{R}}}}/{{\mbox{N}}}}$$

### Electrode preparation and H_2_O_2_ electrosynthesis

The electrodes were prepared by loading as-synthesized TMPc-OCNT catalysts on GDE. In a typical process, 10 mg of catalyst was dispersed in the mixture that consists of 0.8 mL H_2_O, 0.2 mL isopropanol and 50 μL of Nafion (5 wt%) to form catalyst ink. 50 μL of catalyst ink was coated on GDE by hand-painting, followed by drying treatment at 40 ^o^C. The catalyst loading is about 0.5 mg cm^−^^2^. Pt foil was used as the counter electrode to construct a two-electrode system for electrochemical H_2_O_2_ production. The proton exchange membrane (Nafion 117) was used to separate the cathode and anode. The H_2_O_2_ electrosynthesis was carried out at the constant currents using two types of flow-cell electrolyzers shown in Supplementary Figs. 28 and 36. When employing the small reactor with a working area of 1 cm^−^^2^, KOH electrolyte (0.01, 0.1, 0.2, 0.5 or 1.0 M) was used as the catholyte with one-pass flow rate of 5 mL h^−^^1^ to bring out the generated H_2_O_2_, and the anolyte of 0.5 M H_2_SO_4_ was cycled at a rate of 33 mL h^−^^1^. Pure O_2_ was supplied to the cathode side faced to the gas chamber at a flow rate of 20 mL min^−^^1^ that was controlled by a mass flow controller (Sevenstar D07, China). The constant currents were provided by a workstation (Chenhua CHI760E, Shanghai, China). When employing the bigger reactor with a working area of 4 cm^−^^2^, the catholyte was one-time outflowed at a rate of 80–100 mL h^−^^1^, while the anolyte was cycled at the same flow rate. The flow rate of pure O_2_ was controlled at 30 mL min^−1^. The couples of 0.01 M KOH+0.5 M H_2_SO_4_, 0.1 M KOH+0.5 M H_2_SO_4_, 1 M KOH+0.5 M H_2_SO_4_, 1 M KOH+1 M KOH, 0.3 M K_2_SO_4_+0.3 M K_2_SO_4_ (pH 7.2 or 1.5 that is adjusted using H_2_SO_2_) were used as catholyte and anolyte to estimate the performance of H_2_O_2_ electrosynthesis. The constant currents were provided by a direct-current power supply. The H_2_O_2_ concentration was measured by the cerium sulfate titration method based on the stoichiometry of 2 Ce^4+^ + H_2_O_2_ = 2 Ce^4+^ + 2 H^+^ + O_2_. In a typical operation, 10 μL of H_2_O_2_ solution outflowed from the reactor was added into the certain amount of Ce^4+^ solution to reduce Ce^4+^ to Ce^3+^. The Ce^4+^ concentration can be detected by spectrophotometry at 316 nm. Thus, we calculated H_2_O_2_ concentration via the equation of:

7 $$C({{{\mbox{H}}}}_{2}{{{\mbox{O}}}}_{2})=\frac{[{{{\mbox{C}}}}_{0}({{{\mbox{Ce}}}}^{4+})-C({{{\mbox{Ce}}}}^{4+})]\times V({{{\mbox{Ce}}}}^{4+})}{2\times V({{{\mbox{H}}}}_{2}{{{\mbox{O}}}}_{2})}$$

It should be noted that the consumed volume ($V({{{\mbox{Ce}}}}^{4+})$, mL) of Ce^4+^ solution (the initial concentration ${{{\mbox{C}}}}_{0}({{{\mbox{Ce}}}}^{4+})$ is 1 mM) for detecting H_2_O_2_ was at the range of 5–40 mL, which was far larger than$\,V({{{\mbox{H}}}}_{2}{{{\mbox{O}}}}_{2})$ of the H_2_O_2_ solution (10 μL). Otherwise, this simplified equation above needs to be corrected.

### Electricity consumption calculation

The electricity consumption for per kg H_2_O_2_ (3 wt%) production is calculated according to the equation of

8 $$E=\frac{30\times U\times I}{C\times {{\mbox{M}}}({{{\mbox{H}}}}_{2}{{{\mbox{O}}}}_{2})\times v}$$

*E* represents electricity consumption (kWh kg^−^^1^ 3 wt% H_2_O_2_), *I* and *U* represent applied current and cell potential (V, A), respectively, and *C*, M(H_2_O_2_) and *v* represent H_2_O_2_ concentration (mol L^−^^1^), H_2_O_2_ molecular weight (34 g mol^−^^1^) and flow rate, respectively.

### Reporting summary

Further information on research design is available in the Nature Portfolio Reporting Summary linked to this article.

## Supplementary information

Supplementary Information

Peer Review File

Reporting Summary

## Data Availability

The data supporting the conclusions of this study are present in the paper and the Supplementary Information. The raw data sets used for the presented analysis within the current study are available from the corresponding authors upon reasonable request. Source data are provided with this paper.

## Source data

Source Data
