# Supplementary material for: Metal single-site catalyst design for electrocatalytic production of hydrogen peroxide at industrial-relevant currents
Source: Nat Commun. 2023 Jan 12;14:172. doi: 10.1038/s41467-023-35839-z (PMC9837053; doi:10.1038/s41467-023-35839-z)
Supplement: Supplementary file 1 — Supplementary Information [file 41467_2023_35839_MOESM1_ESM.pdf]

Supporting Information

Metal single-site catalyst design for electrocatalytic production of hydrogen peroxide at industrial-relevant currents

Peike Cao[1], Xie Quan[1*], Xiaowa Nie[2], Kun Zhao[3], Yanming Liu[1], Shuo Chen[1], Hongtao Yu[1] & Jingguang G. Chen[4*]

[1]Key Laboratory of Industrial Ecology and Environmental Engineering (Ministry of Education, China), School of Environmental Science and Technology, Dalian University of Technology, Dalian, 116024, PR China

[2]State Key Laboratory of Fine Chemicals, School of Chemical Engineering, Dalian University of Technology, Dalian, 116024, PR China

[3]College of Environmental Science and Engineering, North China Electric Power University, Beijing, 102206, PR China

[4]Department of Chemical Engineering, Columbia University, New York, NY, 10027, USA

*e-mail: quanxie@dlut.edu.cn; jgchen@columbia.edu

Supplementary methods

In situ ATR-SEIRAS. In situ ATR-SEIRAS measurements were on a Bruker Vertex80V IR spectrophotometer with a mercury cadmium telluride (MCT) detector. The working electrode is prepared by a two-step deposition procedure, which includes the chemical deposition of an Au layer on the Si prism followed by catalyst material coating. Specifically, gold plating solution is prepared by the following steps: 1) prepare $AuCl_4^-$ solution by dissolving 0.105 g NaOH in 67 mL ultrapure water followed by adding 3 mL of 0.1 g mL^{-1} $HAuCl_4$ aqueous solution. 2) dissolving 0.1337 g NH_4Cl, 0.6205 g $Na_2S_2O_3 \cdot 5H_2O$, 0.9653 g $Na_2S_2O_3$ in 30 mL ultrapure water in sequence. 3) Mixing the former solution (7 mL) and latter solution (3 mL), and stirring it for 3 h to obtain clear colorless solution. Before Au deposition operation, the Si prism is soaked in 40% NH_4F for 90 s followed by water flushing. The chemical deposition of Au is conducted in a mixture including 2 mL of gold plating solution and 17 μL of 40% HF at 55°C, followed by washing with ultrapure water and ethanol, respectively. Finally, dropping 40 μL of catalyst ink (described in Methods part of main text) on the Au deposited Si prism followed by natural drying to working prepare electrode. As shown in Supplementary Fig. 27, in situ ATR-SEIRAS measurements are conducted in a single-cell reactor with Pt plate and Ag/AgCl electrode being used as the counter and reference electrodes. In situ ATR-SEIRAS for electrocatalytic H_2O_2 synthesis are recorded at different potentials in O_2-saturated 1.0 M KOH by continuously bubbling O_2.

Pollutant degradation and wastewater treatment. We built a flow-cell system combining electrosynthesis of H_2O_2 and the Fenton reaction ($Fe^{2+} + H_2O_2 \rightarrow \cdot OH + OH^- + Fe^{3+}$) for wastewater treatment. Phenol, bisphenol A and levofloxacin are used as the targeted pollutants to evaluate the pollutant removal capacity of the flow-cell Fenton system. The synthetic wastewater containing 1 mM Fe^{2+} and 10 mg L^{-1} pollutants (pH is adjusted to about 3 to prevent iron ion precipitation) is pumped into the Fenton reactor at a rate of about 700 mL h^{-1}. The real coking wastewater with an initial TOC of 44.1 mg L^{-1} is taken from a sewage treatment plant in Dalian, China. The coking wastewater with a dosage of 5 mM Fe^{2+} was pumped into the flow-cell system at a flow rate of 140 mL h^{-1}. We performed total 12 hours

operation in intermittent stages to perform the wastewater treatments. The concentrations of pollutants are measured by the high-performance liquid chromatography (HPLC, HITACHI) equipped with UV detector and C18 chromatographic column (parameters for different pollutants are listed in Supplementary Tab. 9). The total organic carbon (TOC) is analyzed using a multi-N/C 2100S analyzer (Analytik jena).

Supplementary Figures

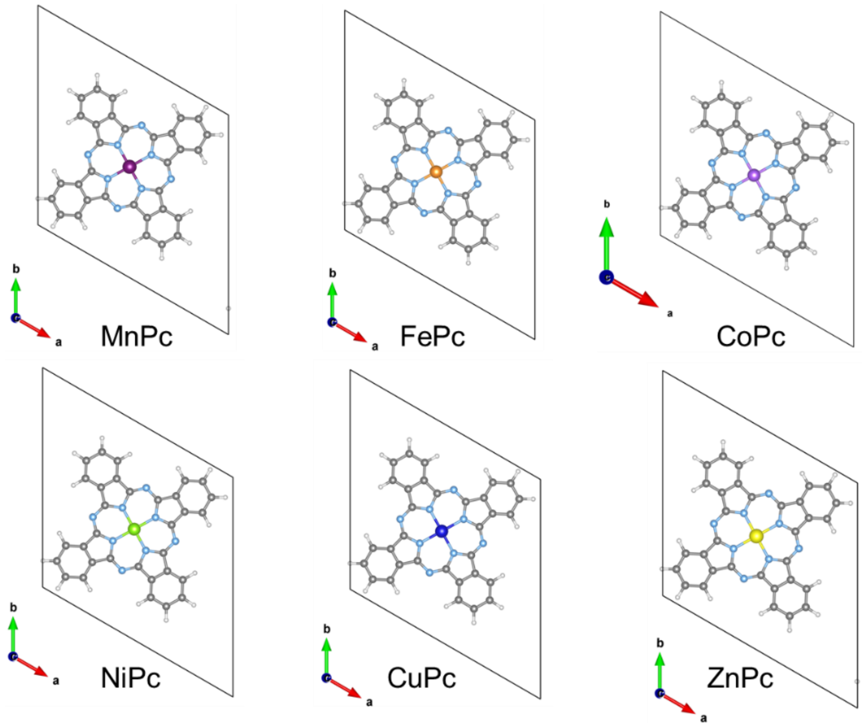

Supplementary Figure 1. Transition-metal phthalocyanine (TMPc) models after DFT optimization. MnPc, FePc, CoPc, NiPc, CuPc and ZnPc. The dark magenta, orange, violet, shiny green, blue and yellow spheres for Mn, Fe, Co, Ni, Cu and Zn atoms, respectively. The dark gray, sky blue and grey white spheres for C, N and H atoms, respectively.

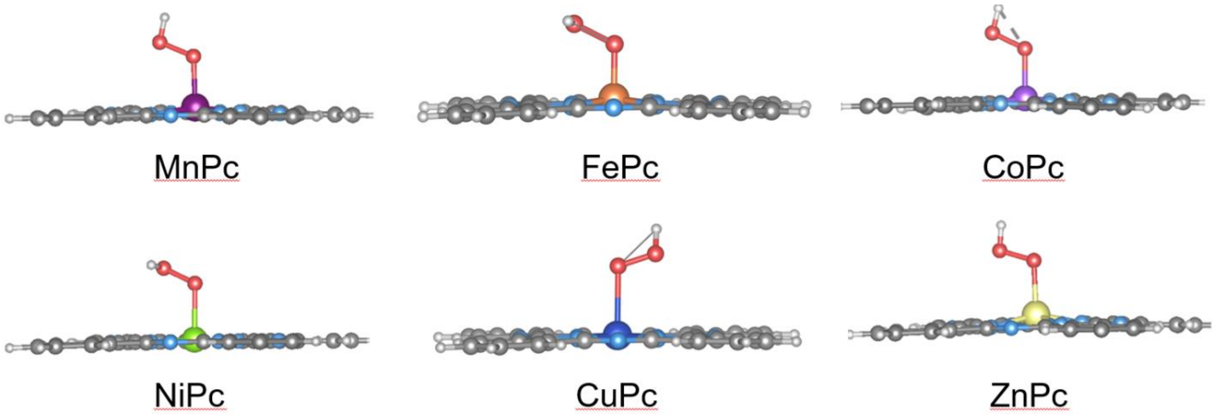

Supplementary Figure 2. The configurations of *OOH adsorbed on the TM sites of TMPc after DFT optimization. The dark magenta, orange, violet, shiny green, blue and yellow spheres for Mn, Fe, Co, Ni, Cu and Zn atoms, respectively. The dark gray, sky blue and grey white spheres for C, N and H atoms, respectively.

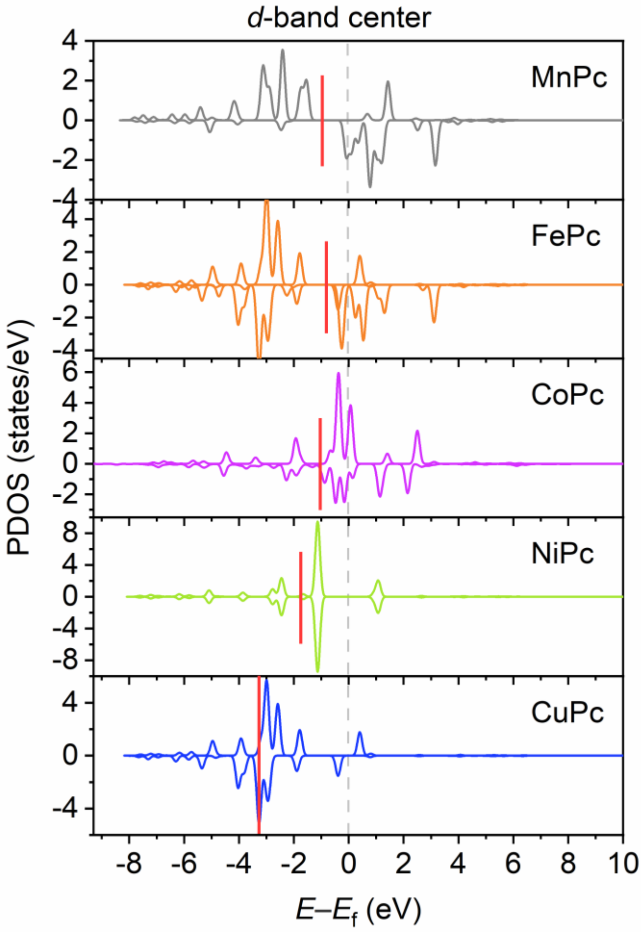

Supplementary Figure 3. Projected density of states (PDOS) for *d*-orbitals of TM atoms of TMPc. The red lines mark the positions of d-band center.

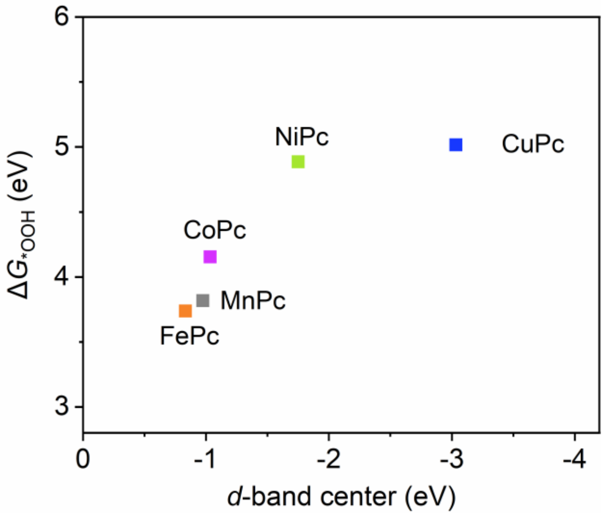

Supplementary Figure 4. The relationship between the *OOH adsorption energy (ΔG_{*OOH}) of TMPc and the d-band center.

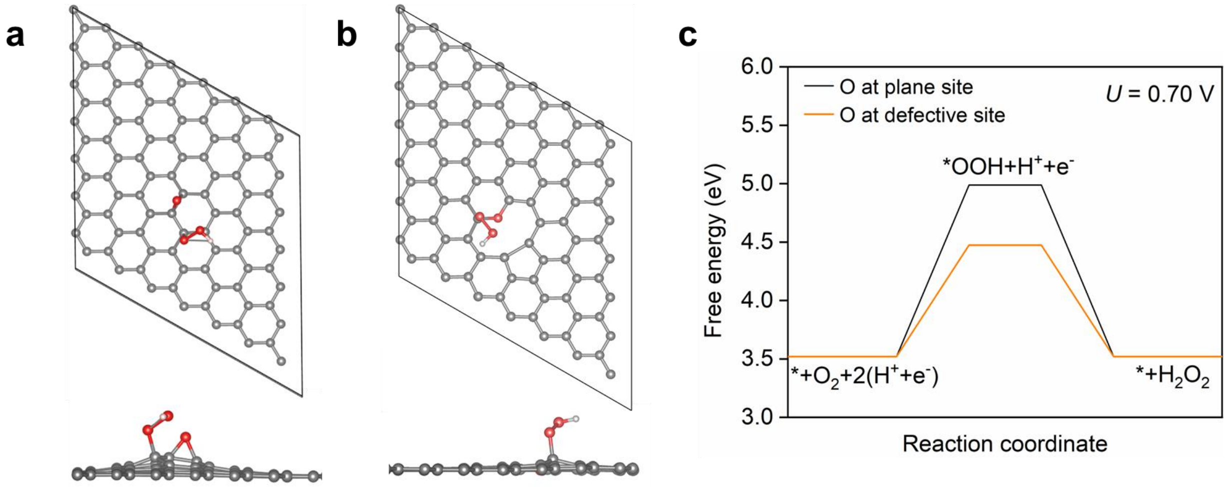

Supplementary Figure 5. The *OOH adsorption estimation of OCNT. The adsorption of *OOH on OCNT with O atom at **a,** plane site and **b,** defective site. The red, dark gray and grey white spheres for O, C and H atoms, respectively. **c,** Calculated free energy diagrams of two-electron ORR on OCNT.

Supplementary Note 1: The O modified graphitic carbon show poor *OOH binding abilities and high energy barrier for *OOH formation, indicating that their activity contributions are not crucial for ORR process on CoPc-OCNT.

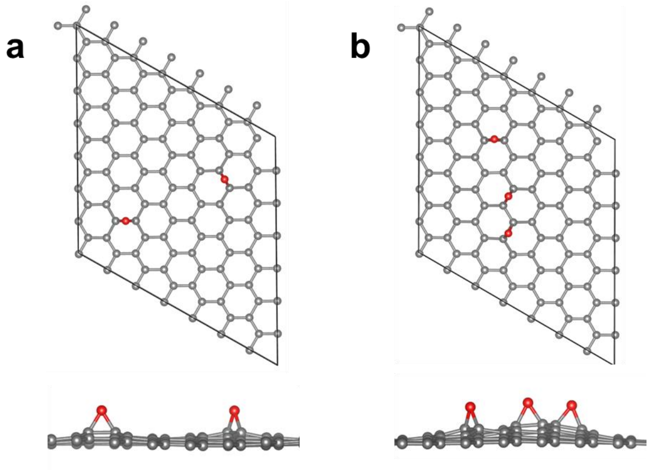

Supplementary Figure 6. Other possible OCNT models after DFT optimization. a, Two or **b,** three O atoms are introduced on the graphite plane.

Supplementary Note 2: We consider the possible cases that two or three O atoms are introduced on the carbon plane to coordinate with the Co center of CoPc; however, none of the stable configurations are achieved. The red, dark gray and grey white spheres for O, C and H atoms, respectively.

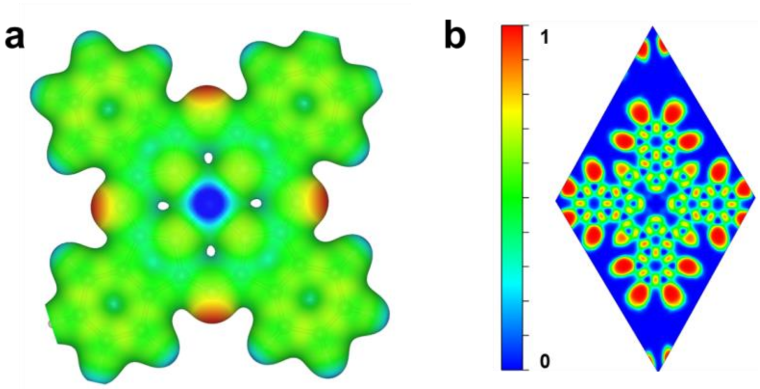

Supplementary Figure 7. Electrostatic potential analysis of CoPc. a, Electrostatic potential with isosurface level of 0.02 e/Bohr^3. **b,** Electron localization function**.**

Supplementary Note 3: Electrostatic potential shows that the blue region has a higher electrostatic potential, which is more likely to be attacked by nucleophilic reagent such as O_2 molecule. The range of electron localization function values varies from 0 to 1 that the value of 1 is for the high localization (red color area), and the value of 0 indicates the zero localization (blue color area). The Co center presents low electron localization that is the characteristic of electrovalent bond, which can be regarded as the nucleophilic attack sites. Thus, it is demonstrated that the Co center is ORR active due to the high electrophilicity.

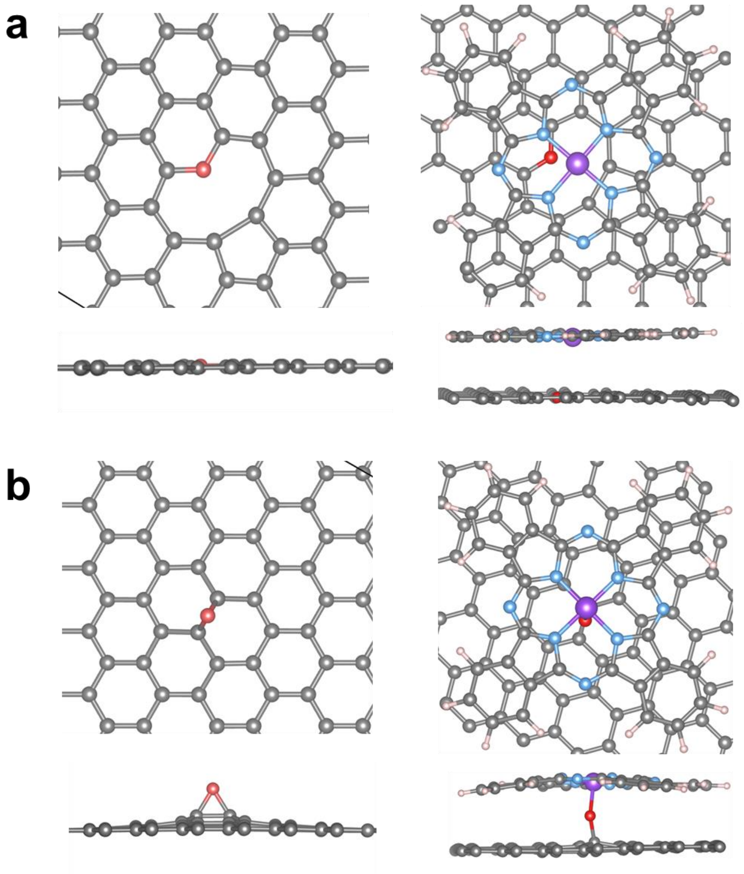

Supplementary Figure 8. Two types of OCNT models and their supported CoPc. a, O atom at the defective site to coordinate Co center of CoPc (denoted as CoPc-OCNT). **b,** Axial O atom on the carbon plane to coordinate Co center of CoPc (denoted as CoPc/OCNT). The violet, sky blue, red, dark gray and grey white spheres for Co, N, O, C and H atoms, respectively.

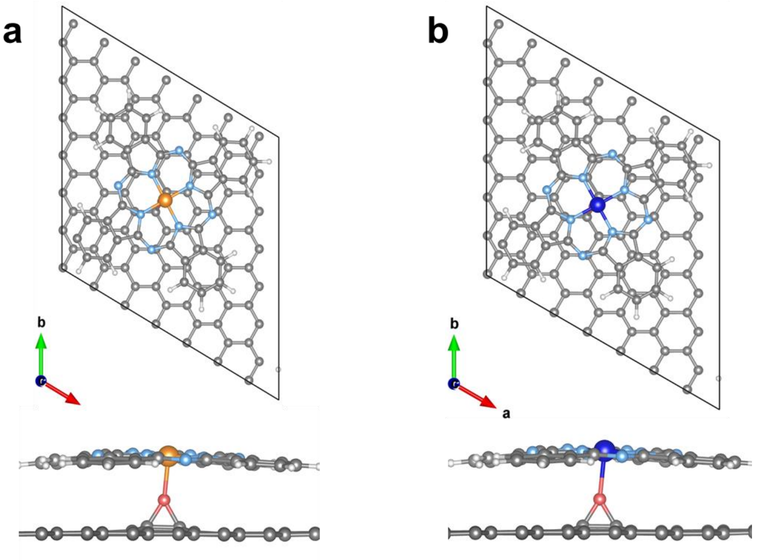

Supplementary Figure 9. Top and side views of FePc and CuPc supported by OCNT. a, FePc/OCNT. **b,** CuPc/OCNT. The orange, blue, sky blue, red, dark gray and grey white spheres for Fe, Cu, N, O, C and H atoms, respectively.

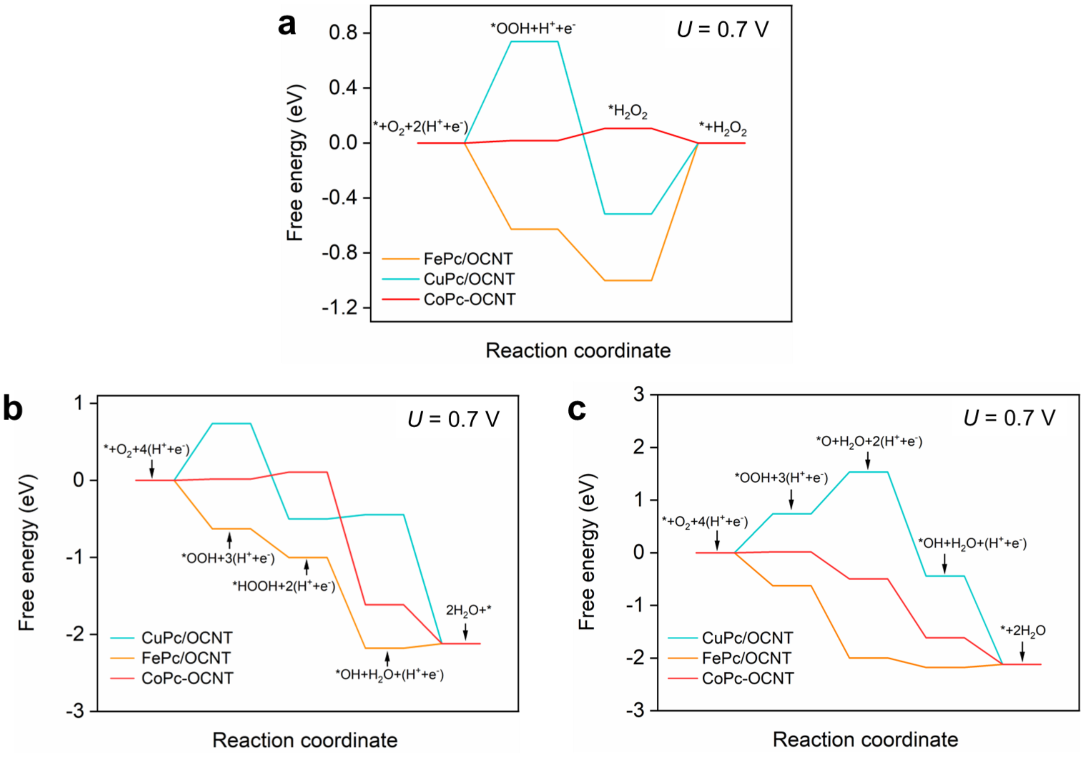

Supplementary Figure 10. Calculated free-energy diagram for two-electron ORR process. a, Free energy diagrams of two-electron ORR toward H_2O_2 formation on CoPc-OCNT, FePc/OCNT and CuPc/OCNT. Free energy diagrams of four-electron ORR on CoPc-OCNT, FePc/OCNT and CuPc/OCNT: **b,** the disproportionation of H_2O_2 and **c,** the dissociation of *OOH pathways.

Supplementary Note 4: The free energy diagrams of two-electron ORR at 0.7 V exhibit a large energy barrier to form *OOH on the CuPc-OCNT catalyst, which suggests a thermodynamically unfavorable ORR processes and low ORR activity due to the poor adsorption of oxygen-containing intermediates. The large uphill energy barrier to desorb *H_2O_2 to generate the H_2O_2 product on the FePc/OCNT catalyst suggests a poor H_2O_2 selectivity. The CoPc-OCNT catalyst has a small energy barrier during the entire process, indicating a low overpotential of two-electron ORR. These results from free energy diagrams are consistent with the activity-volcano curve of the two-electron ORR based on the *OOH binding energy.

We further examine the H_2O_2 selectivity of CoPc-OCNT by inspecting the O–O bond dissociation in the adsorbed *OOH or *H_2O_2. Although the free energy diagrams of the four-electron ORR of CoPc-OCNT show more thermodynamically favorable O–O bond dissociation via the dissociation of *H_2O_2 or *OOH over H_2O_2 formation, it might incorrectly imply a low H_2O_2 selectivity due to the potentially higher kinetic energy barrier of O–O bond breakage.

On the question why many catalysts, despite the strong thermodynamic preference for the O–OH breaking, exhibit high selectivity for H_2O_2 production. Previous microkinetic modeling studies[1, 2] indicate that kinetic parameters have a high degree of rate control and are essential for describing the selectivity. Liu and collaborators [3, 4] recently reported an advanced first-principles model for the effective calculation of the electrochemical reaction kinetics at the solid−water interface. They found that the initial stretching of the O−OH bond on the single cobalt atom catalyst could induce an energy barrier higher than the breaking of *−O, thus preferring the H_2O_2 formation. Decreasing potential promotes proton adsorption to the O in *−O−OH, thereby increasing the H_2O_2 selectivity of the single cobalt atom catalyst, while for the carbon catalyst, the proton prefers the latter O, resulting in a lower H_2O_2 selectivity in acidic condition.

Thus, the purely thermodynamic analysis of the free energy diagrams of reaction coordinate is likely insufficient to capture the experimentally observed trends in ORR selectivity that might be attributed to kinetics paraments.

It is worth mentioning that the descriptor of *OOH binding energy is still effective to preliminarily screen the two-electron ORR catalysts, as an optimal *OOH binding determines for the steps of *O_2→*OOH and *OOH→*H_2O_2.

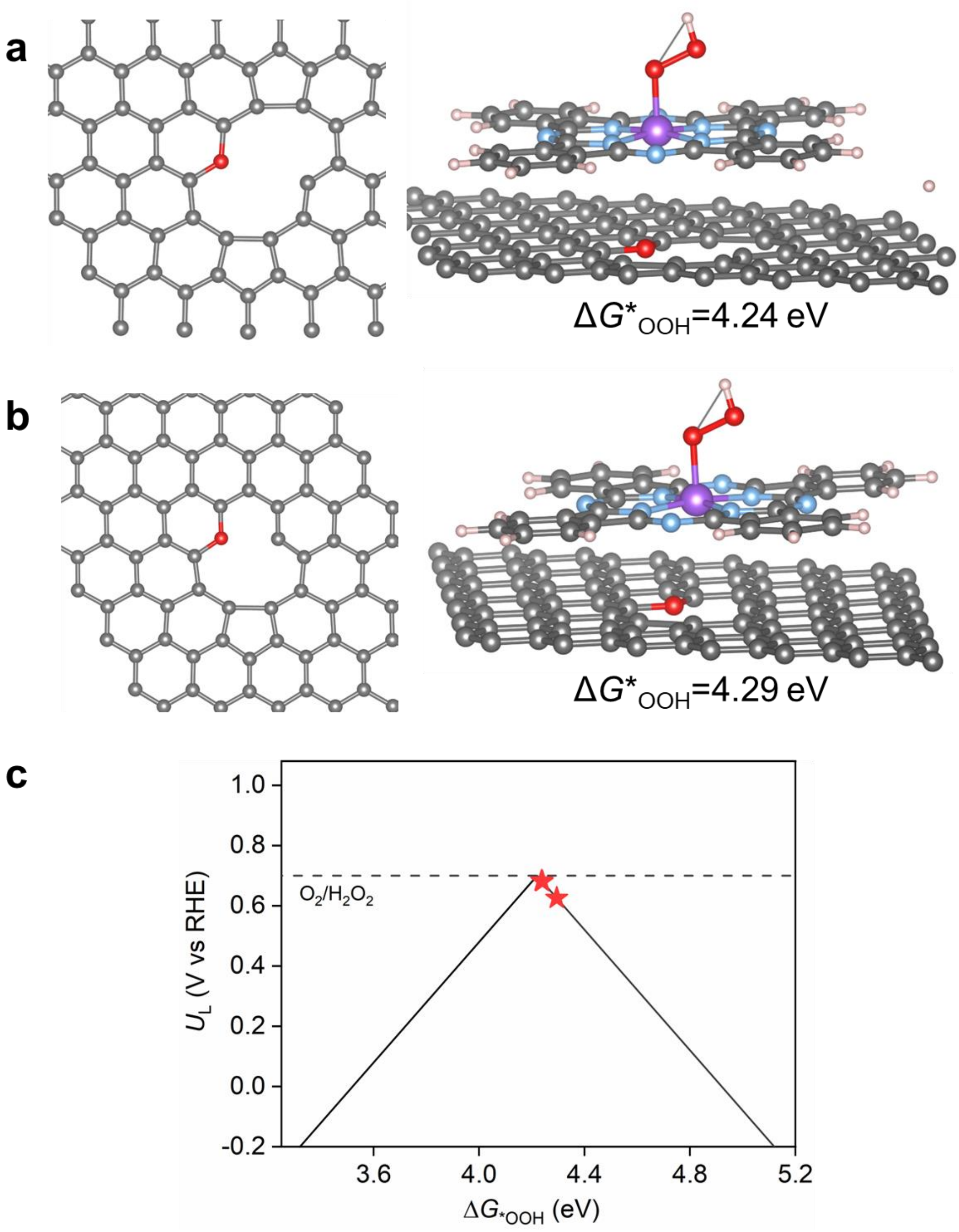

Supplementary Figure 11. The performance estimation of CoPc supported by different types of O substituting C in different defective sites. a-b, Atomic structures of OCNT supported CoPc. **c,** The calculated ΔG_{*OOH} in the activity-volcano curve of two-electron ORR.

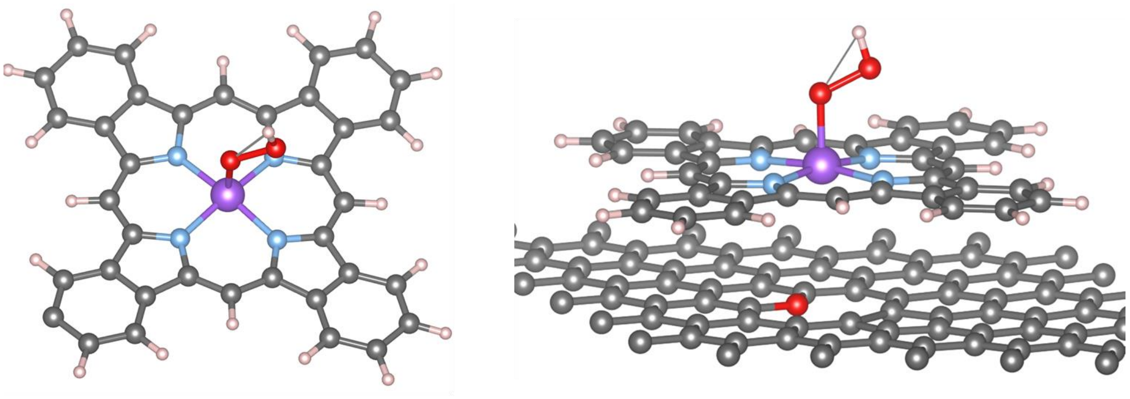

CoTBP(ΔG^*_{OOH}=4.09 eV) CoTBP-OCNT(ΔG^*_{OOH}=4.17 eV)

Supplementary Figure 12. *OOH adsorption on cobalt tetrabenzoporphyrin (CoTBP) and CoTBP-OCNT.

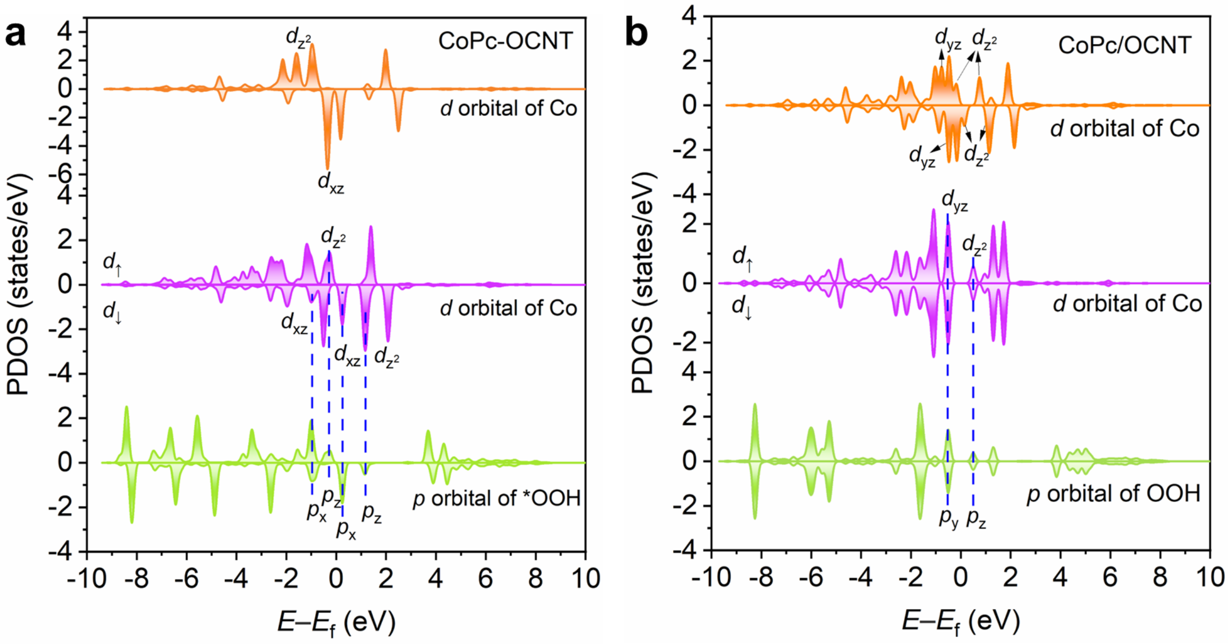

Supplementary Figure 13. Projected density of states (PDOS) for *d*-orbitals of Co atom before (orange) and after (violet) *OOH adsorption, and *p*-orbitals of *OOH (green). a, CoPc-OCNT. **b**, CoPc/OCNT.

Supplementary Note 5: The $d_{\uparrow}$ and $d_{\downarrow}$ represent *d*-orbitals in the states of spin up and spin down, respectively. The hybridized orbitals near the Fermi level are mainly involved in the ORR reactions. The CoPc/OCNT with adsorbed *OOH shows the symmetric PDOS that is contributed by $d_z{}^2$ and d_{yz} coupled with p_z and p_y, respectively, while CoPc-OCNT shows the spin-polarized states composed of $d_z{}^2$ and d_{xz} coupled with p_z and p_x. The significant changes in the PDOS of CoPc/OCNT and CoPc-OCNT before and after *OOH adsorption indicate their different orbital hybridization, which has an important impact on the properties of *OOH adsorption on Co sites.

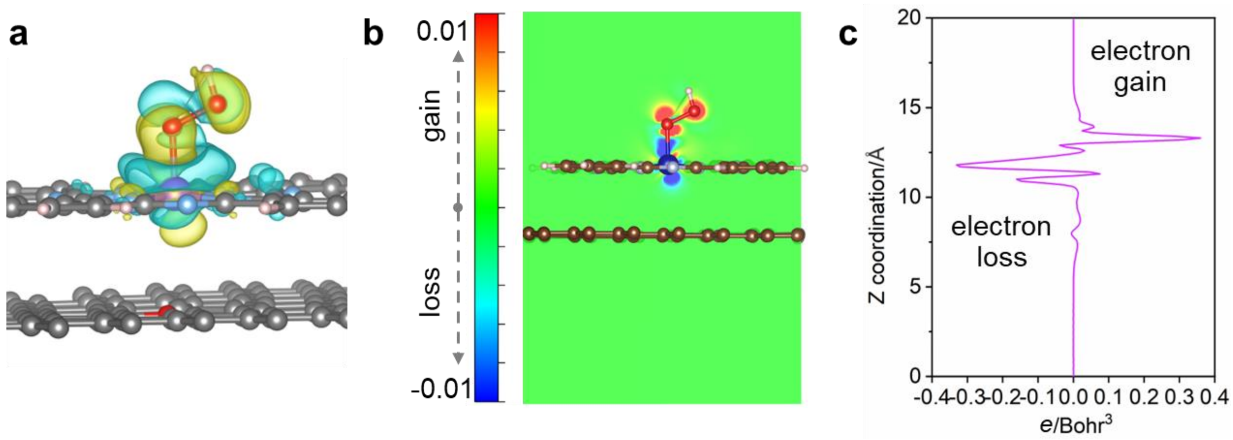

Supplementary Figure 14. Bader charge density difference after *OOH is adsorbed on CoPc-OCNT. a, Charge density difference (cyan and yellow isosurfaces with the level of ±0.001 Bohr^{-3} represent electron loss and electron gain, respectively). **b,** Two-dimensional slice mapping of charge density difference. **c,** Charge integration in Z coordination.

Supplementary Note 6: Charge density difference and charge integration in Z coordination show charge loss at the Co site and charge accumulation at the O atom, suggesting charge transfer from the Co center to O atoms after *OOH is adsorbed.

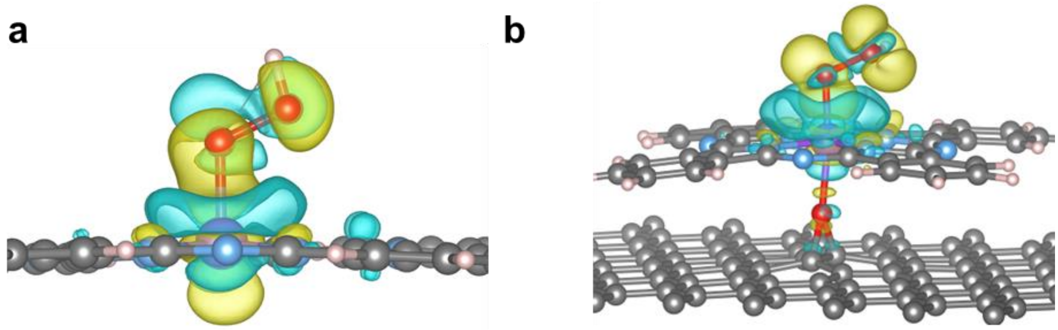

Supplementary Figure 15. Bader charge density difference after *OOH is adsorbed on a, CoPc and b, CoPc/OCNT. Cyan and yellow isosurfaces with the level of ±0.001 Bohr^{-3} represent electron loss and electron gain, respectively.

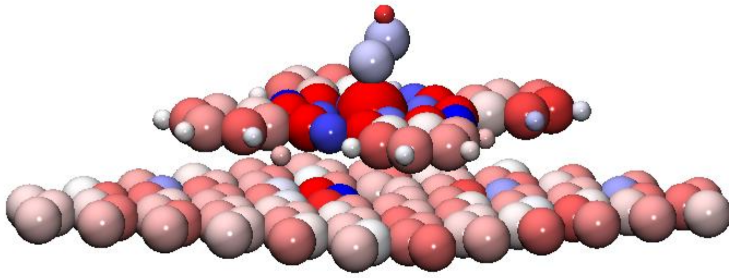

Supplementary Figure 16. Atom charge distribution of CoPc-OCNT. Electrons loss on red atoms and electrons gain on blue atoms.

Supplementary Note 7: It shows charge loss at the Co site and charge gain at adjacent N atoms, suggesting the intrinsic electron-deficient Co center induced by electron-withdrawing N or O coordination. More charge lost on Co atom in CoPc-OCNT (–0.898 |e|) compared with CoPc (–0.898 |e|) and CoPc/OCNT (–0.612 |e|) suggests the intensified electron transfer from Co to surrounding ligands (see Supplementary Table 1), which might be responsible for the weaker *OOH binding on CoPc-OCNT.

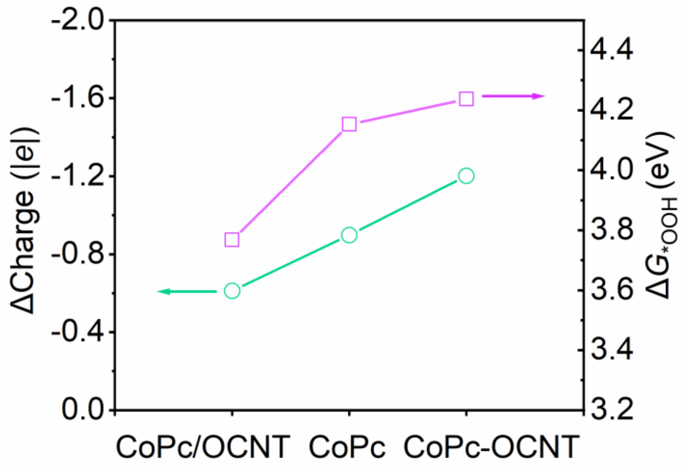

Supplementary Figure 17. The relationship between relative charge difference of Co atom and calculated ΔG_{*OOH}.

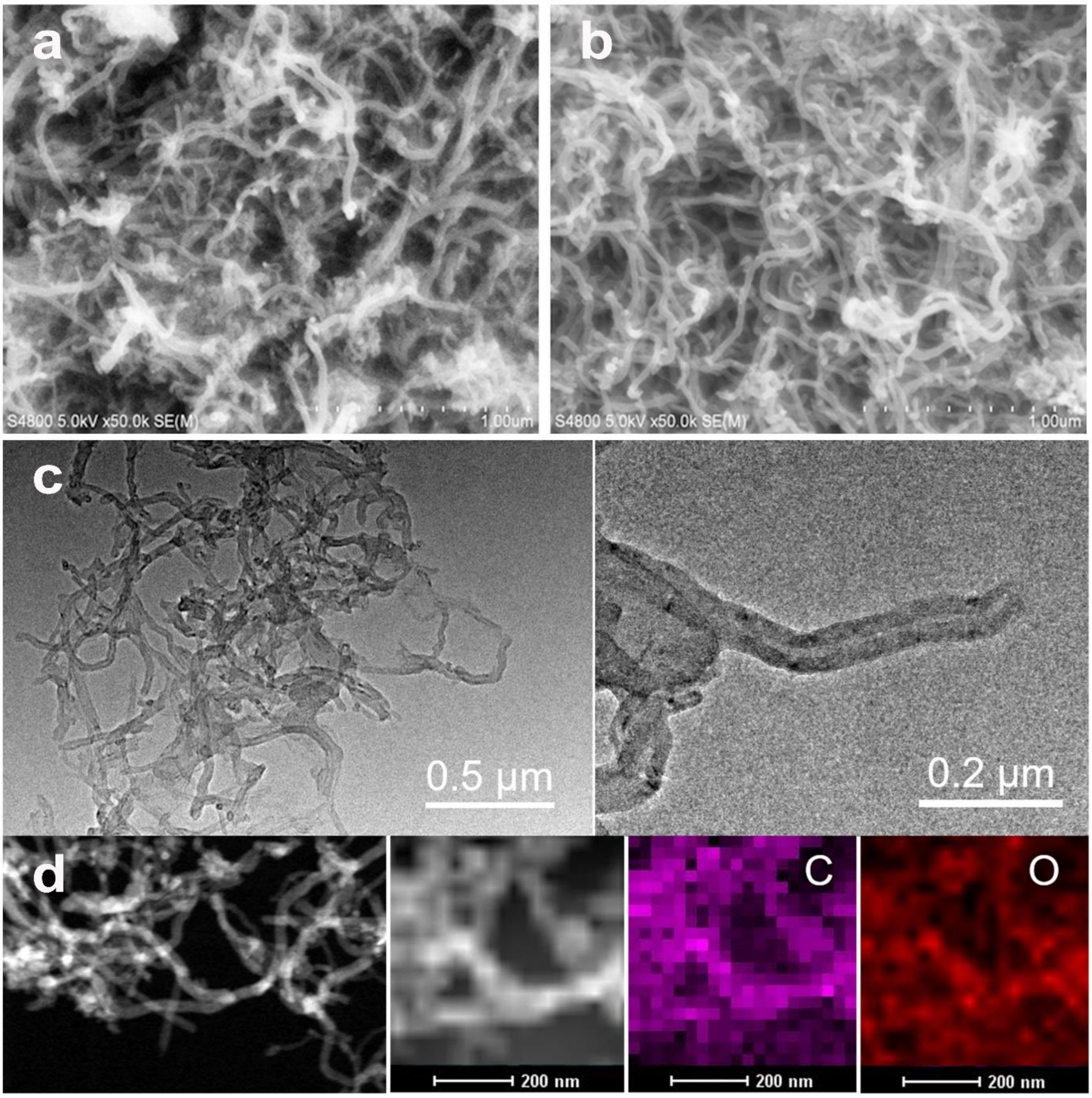

Supplementary Figure 18. Morphology characterization of original CNT (ori-CNT) and OCNT. a, SEM image of ori-CNT. OCNT characterization: **b,** SEM image. **c,** TEM images. **d,** C and O element mapping acquired by energy dispersive spectroscopy.

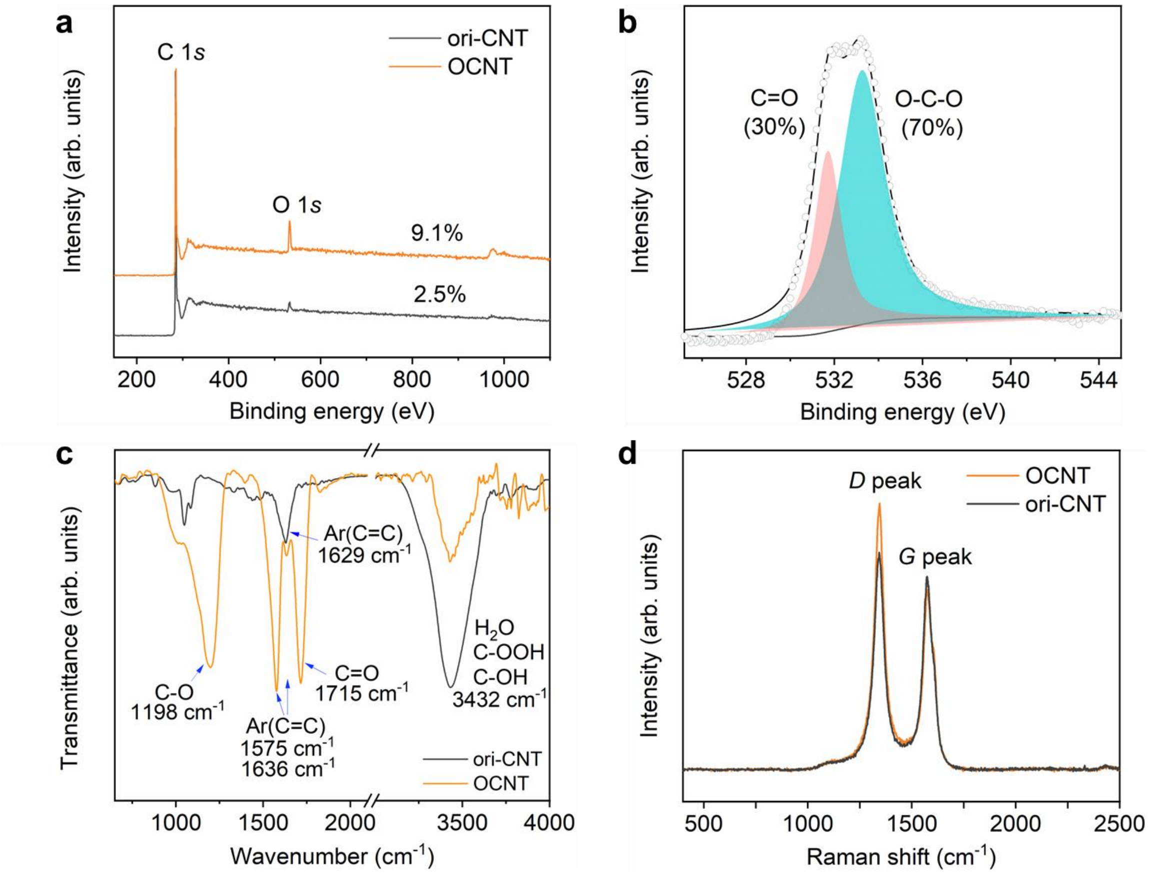

Supplementary Figure 19. Characterization of oxygen functional groups on original CNT (ori-CNT) and OCNT. a-b, XPS survey spectra and O 1*s* high-resolution spectrum of OCNT. **c,** FT-IR spectra. **d,** Raman spectra, the greater *D* peak integration suggests more carbon defect.

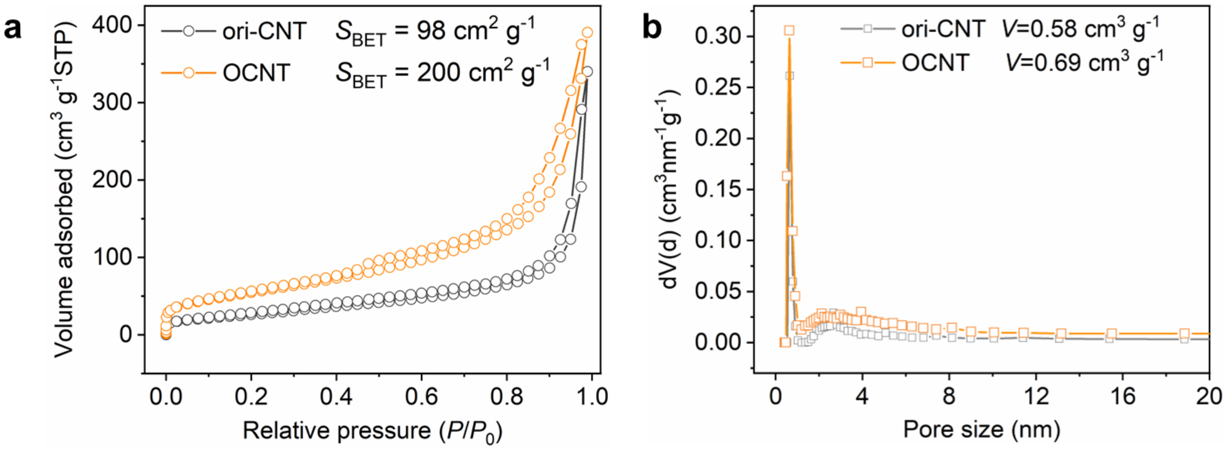

Supplementary Figure 20. Pore characteristics of original CNT (ori-CNT) and OCNT. a, N_2 adsorption-desorption curves (S_{BET} represents the specific surface area calculated using the Brunauer-Emmett-Teller (BET) method). **b,** Pore size distribution curves (*V* represents total volume).

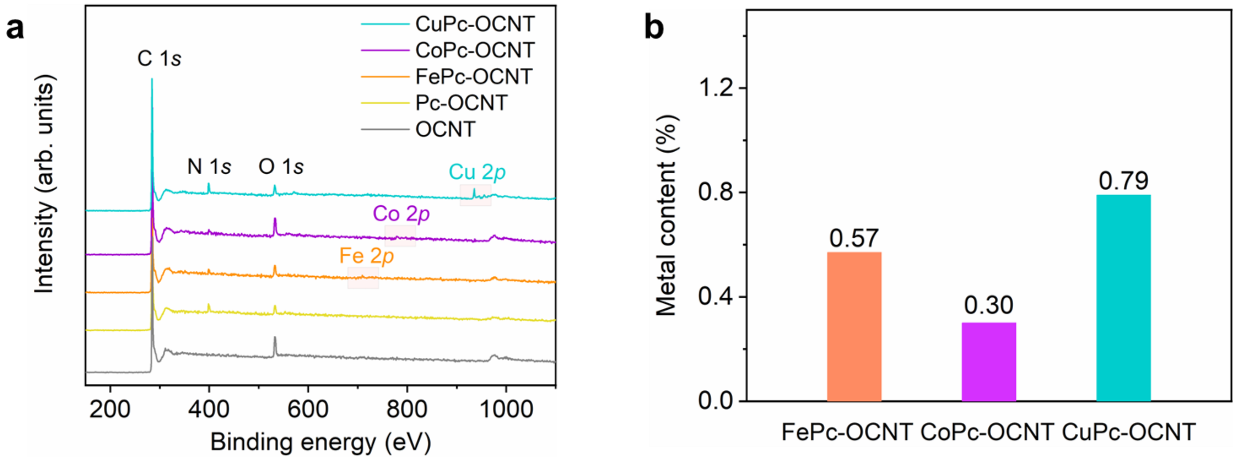

Supplementary Figure 21. Elemental constitutes of TMPc-OCNT catalysts. a, XPS full-survey spectra. **b,** Metal contents of TMPc-OCNT catalysts.

Supplementary Note 8: The XPS survey scans detect the signals assigned to C, N, O, Fe, Co and Cu on the surface of TMPc-OCNT while only C and O on OCNT, indicating the successful loading of TMPc on the OCNT support. The atomic contents of Fe, Co and Cu are determined to be 0.57, 0.30 and 0.79 at% on the surface of FePc-OCNT, CoPc-OCNT and CuPc-OCNT, respectively, while concentrations of these elements in unmodified OCNT are below the detection limit. Apart from XPS, we also used inductively coupled plasma-mass spectrometry (ICP-MS) to analyze the bulk metal loading of catalysts. CoPc-OCNT, FePc-OCNT and CuPc-OCNT has a 0.59 wt% Co, 2.93 wt% Fe or 0.78 wt% Cu loading, respectively. The fact has to be pointed out that we still can detect Fe (~1.100 wt%), Co (~0.007 wt%), Ni (~0.140 wt%) and Cu (~0.037 wt%) in OCNT by inductively coupled plasma-mass spectrometry (ICP-MS) in spite of repeated intense acid leaching, however, the residual metals hardly be detected by XPS (below the detection limit). It can be considered that the residual metals might be buried or sealed in the interior of CNT where cannot be reached by corrosive acid even with the continuous heating and rigorous stirring. Thus, it is reasonable to consider that these remaining metals are hard to be involved in catalytic reactions. We use the metal contents on the surface of TMPc-OCNT measured by XPS to represent the metal loadings.

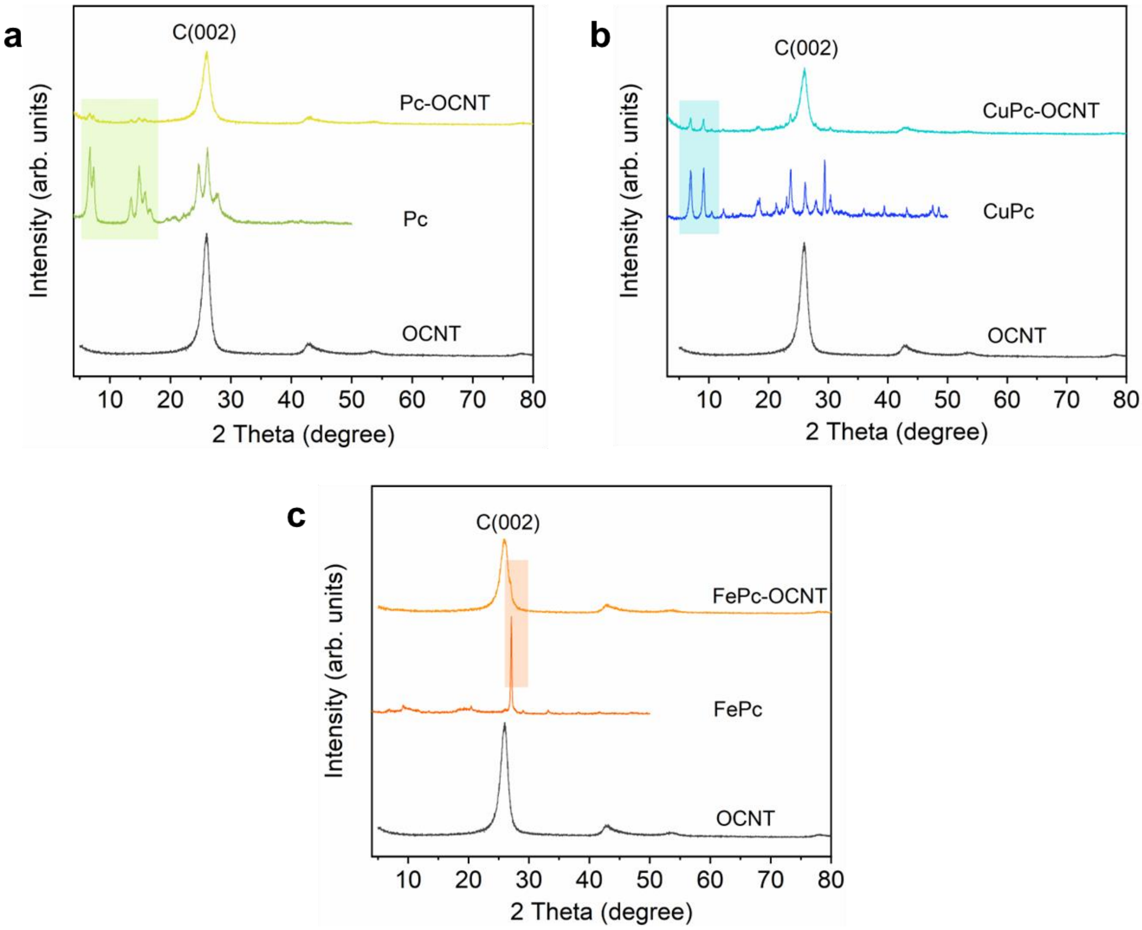

Supplementary Figure 22. Phase structure identification by XRD measurements. a, Pc-OCNT. **b,** CuPc-OCNT. **c,** FePc-OCNT. *C*(002) represents the (002) lattice plane of graphitic carbon.

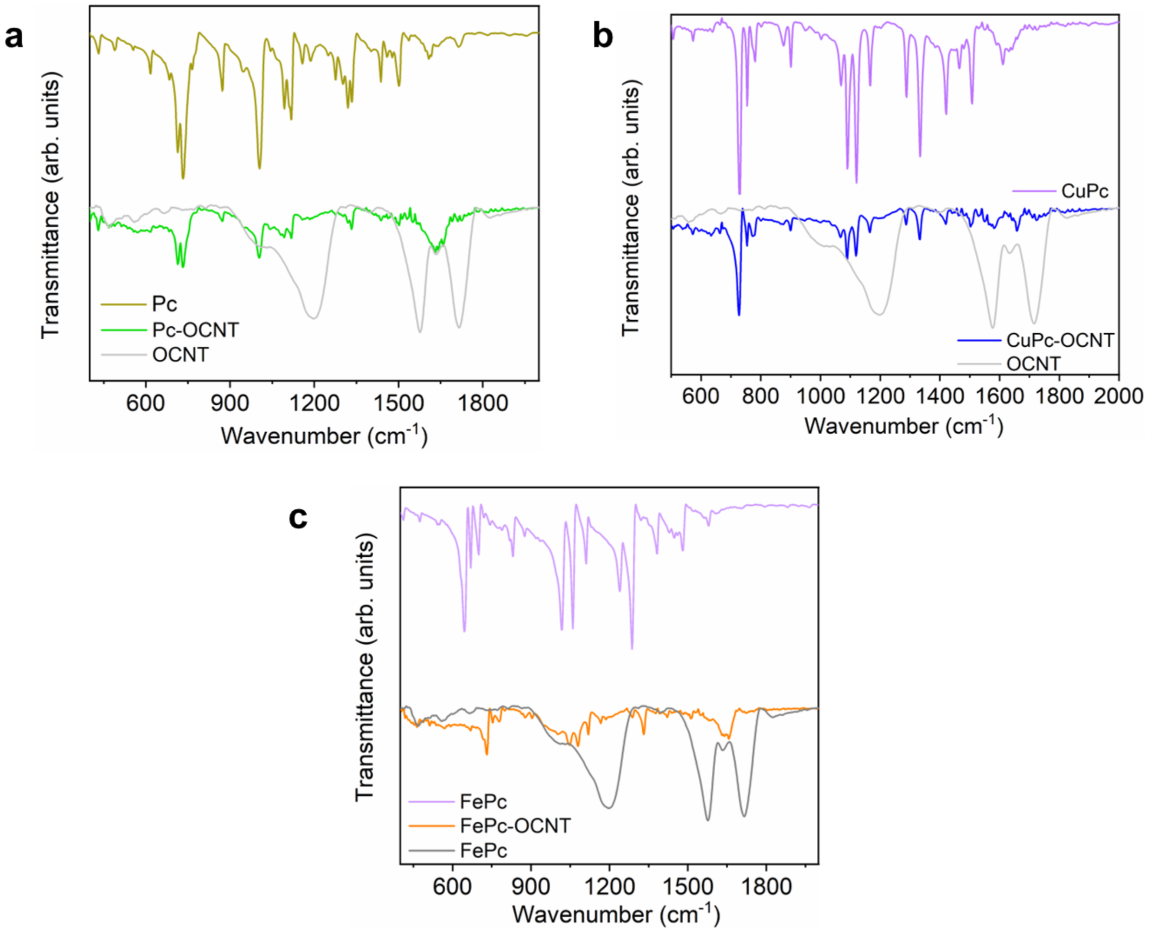

Supplementary Figure 23. Functional group identification by FT-IR spectra. a, Pc and Pc-OCNT. **b,** CuPc and CuPc-OCNT. **c,** FePc and FePc-OCNT. The peaks at 435–873 cm^{-1} correspond to the out-of-plane bending of C–H/C–C in benzene ring, and the peaks at 1090–1524 cm^{-1} can be assigned to the C–H and C–N stretching (more information in Supplementary Table 4).

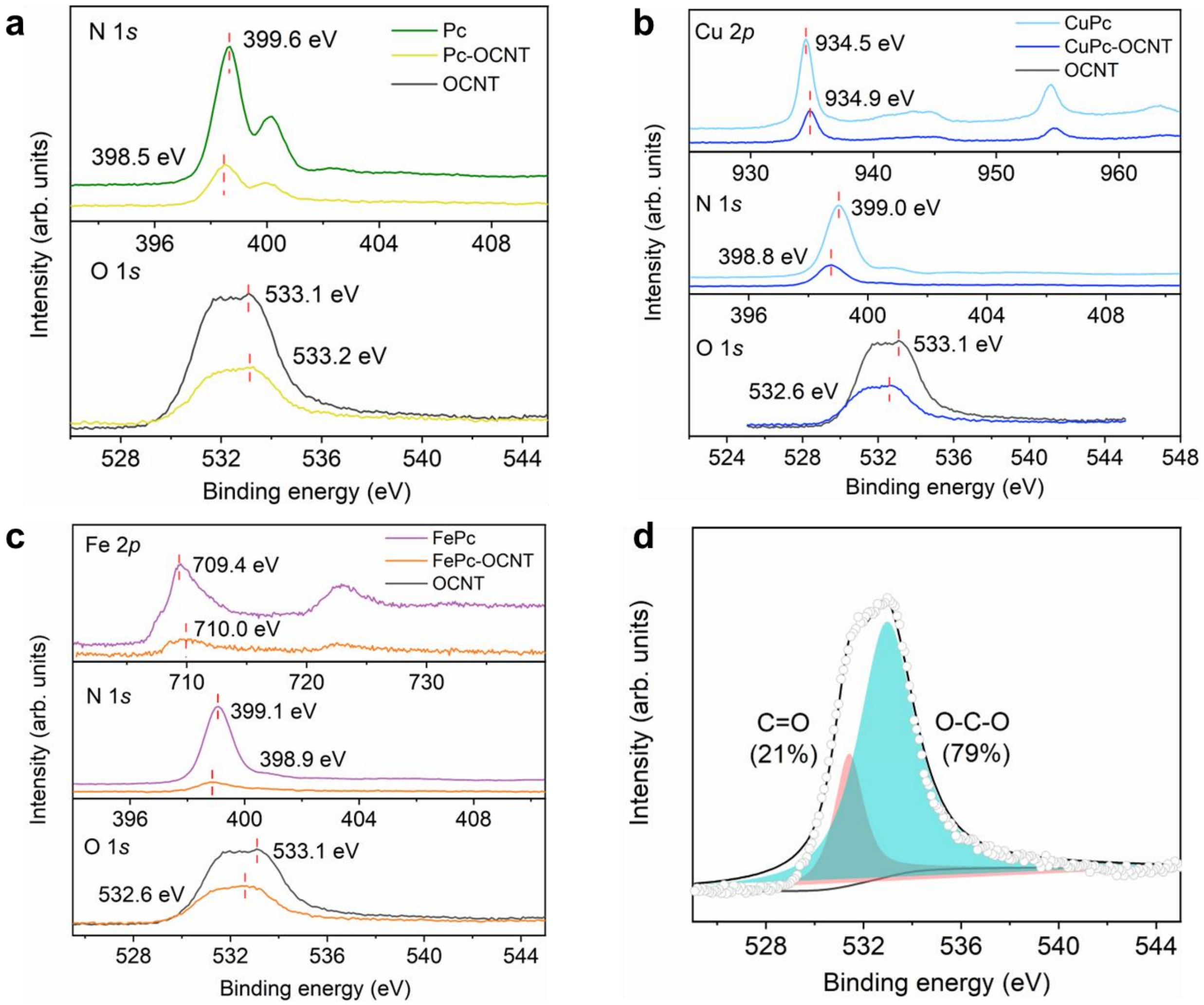

Supplementary Figure 24. XPS high-resolution spectra. a, Pc and Pc-OCNT. **b,** CuPc and CuPc-OCNT. **c,** FePc and FePc-OCNT. **d,** O 1*s* high-resolution spectrum of CoPc-OCNT.

Supplementary Note 9: N 1*s* high-resolution spectra indicate that the N species in CoPc, CoPc-OCNT, CuPc, CuPc-OCNT, FePc and FePc-OCNT mainly exist in the forms of pyridinic-N (398.4 eV) and pyrrolic-N (399.6 eV)[5], which well agrees with their intrinsic molecular structures.

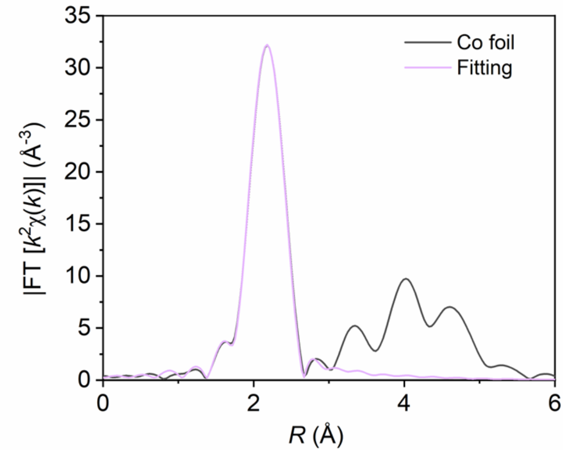

Supplementary Figure 25. FT-EXAFS curve at *R* space for Co K-edge of Co foil and the fitting curve.

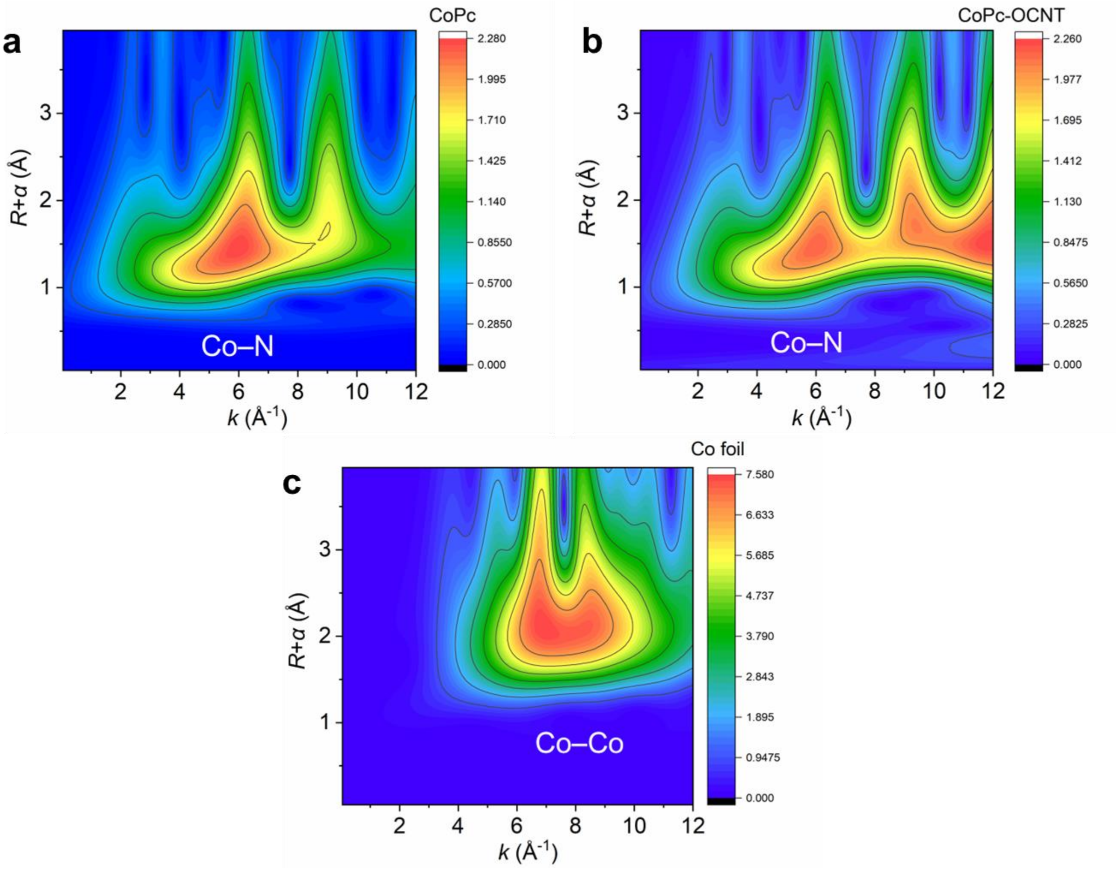

Supplementary Figure 26. WT-EXAFS of Co K-edge. a, CoPc-OCNT. **b,** CoPc. **c,** Co foil.

Supplementary Note 10: The wavelet transform EXAFS (WT-EXAFS) can reflect the bond distance of coordination atoms in the *R* space. The CoPc-OCNT shows a similar WT-EXAFS contour map to that of CoPc, confirming the double-shell coordination of the Co center. The absence of the corresponding peak for the Co–Co bond (centered at (6.93 Å^{-1}, 2.14 Å) in Co foil) confirms the atomic dispersion of Co site in CoPc-OCNT.

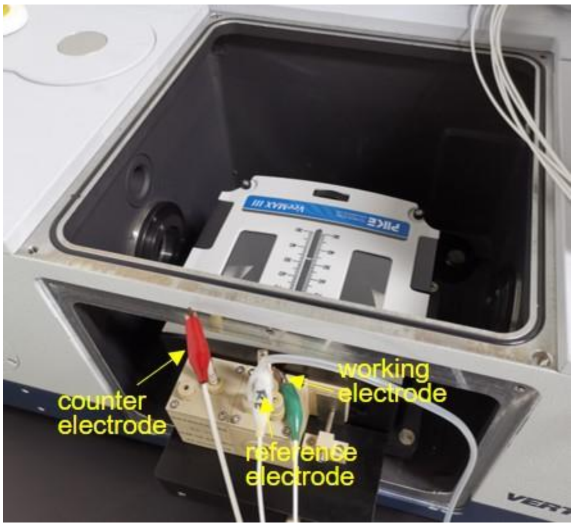

Supplementary Figure 27. Digital photograph showing in situ ATR-SEIRAS set-up for electrocatalytic H_2O_2 synthesis.

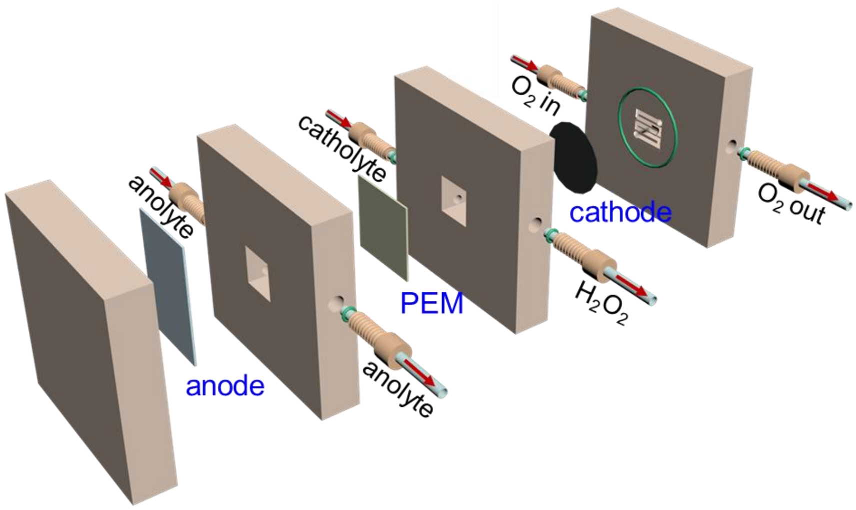

Supplementary Figure 28. Simple drawing of the flow-cell electrolyzer for H_2O_2 electrosynthesis. Test conditions: the working area of 1 cm^{-2}, Pt foil was used as the anode and the proton exchange membrane (PEM) was used to separate the cathode and anode. KOH electrolyte (0.01, 0.1, 0.2, 0.5 or 1.0 M) was used as the catholyte with one-pass flow rate of 5 mL h^{-1} to bring out the generated H_2O_2, and the anolyte of 0.5 M H_2SO_4 was cycled at a rate of 33 mL h^{-1}. Pure O_2 was supplied to the cathode side faced to the gas chamber at a flow rate of 20 mL min^{-1} that was controlled by a mass flow controller (Sevenstar D07, China). The constant currents were provided by a workstation (Chenhua CHI760E, Shanghai, China).

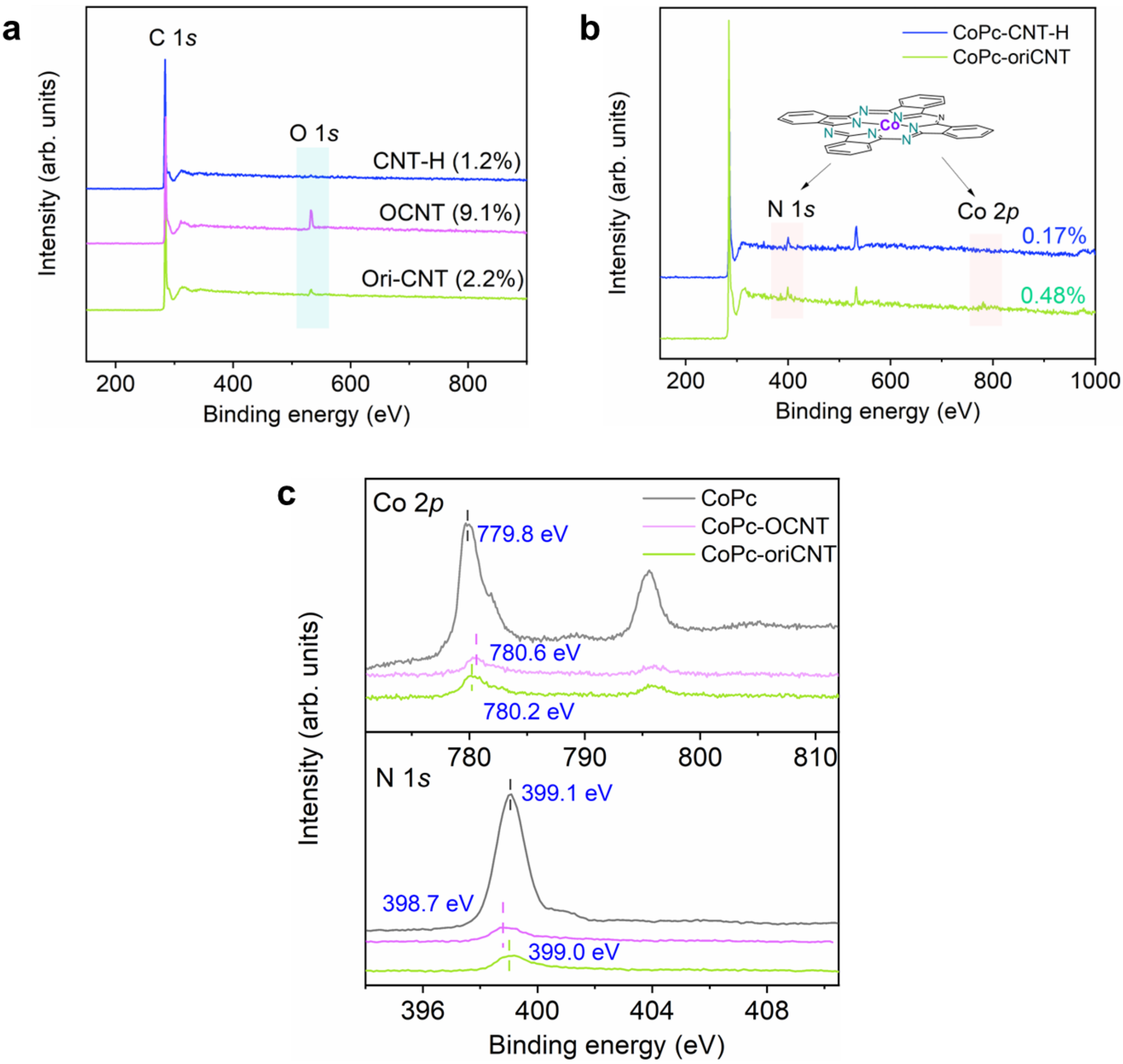

Supplementary Figure 29. XPS spectra. XPS survey spectra and O 1s high-resolution spectrum**. a,** XPS survey spectra of CNT-H catalyst compared with ori-CNT and OCNT. **b,** XPS survey spectra of CoPc-oriCNT and CoPc-CNT-H catalysts (The numbers in figure represent the Co contents). **c,** Co 2*p* and N 1*s* high-resolution XPS spectra of CoPc-oriCNT compared with those of CoPc-OCNT and CoPc.

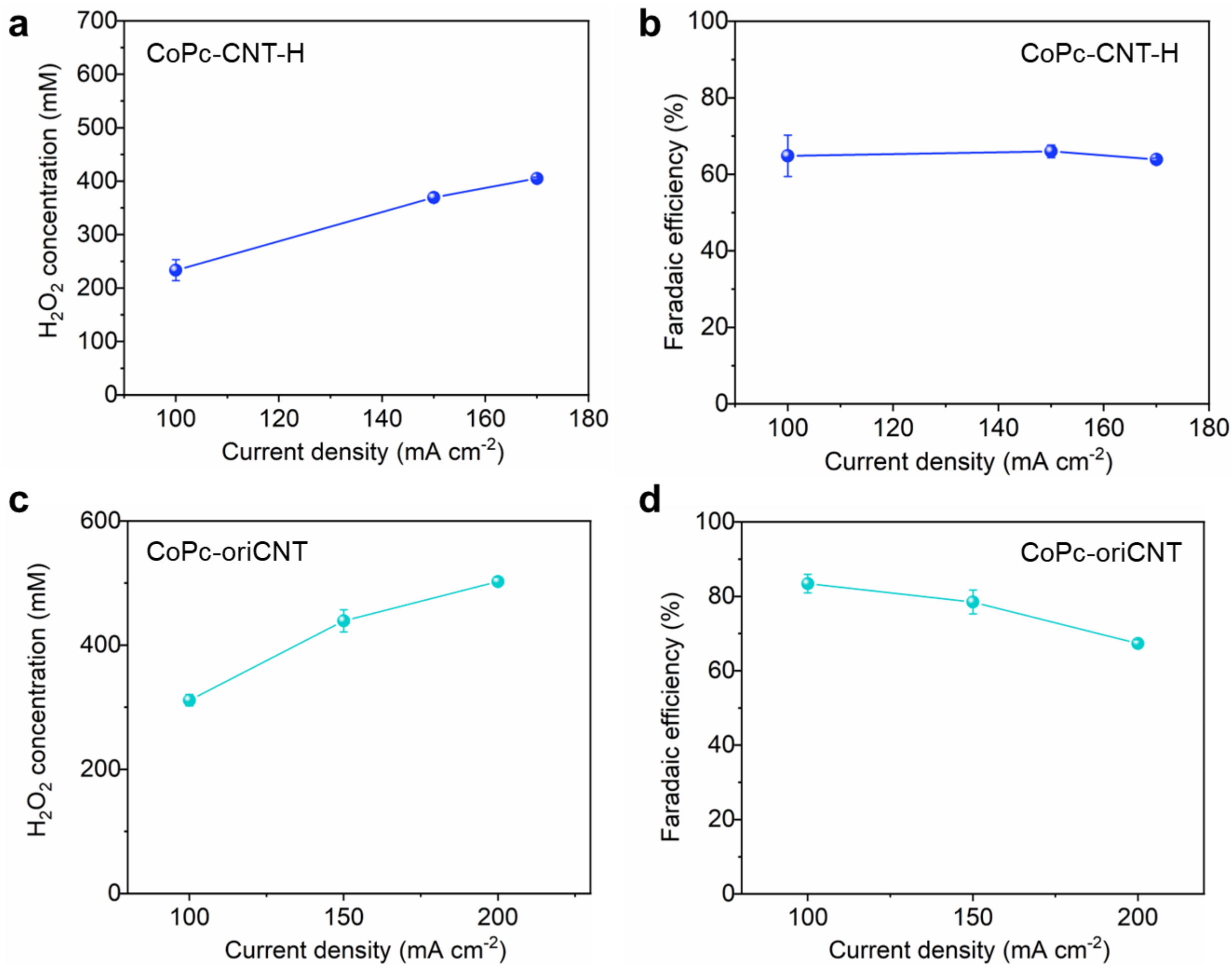

Supplementary Figure 30. The performance of H_2O_2 electrosynthesis on CoPc-CNT-H and CoPc-oriCNT catalysts. a-c, The H_2O_2 concentration. **b-d,** Faradaic efficiency for producing H_2O_2. Experimental conditions: 1.0 M KOH (pH 13.7) was used as the catholyte with one-pass flow rate of 5 mL h^{-1}, and the anolyte of 0.5 M H_2SO_4 was circled at a rate of 33 mL h^{-1} (Data points and error bars represent the average and standard deviation of data from triplicate parallel tests, respectively).

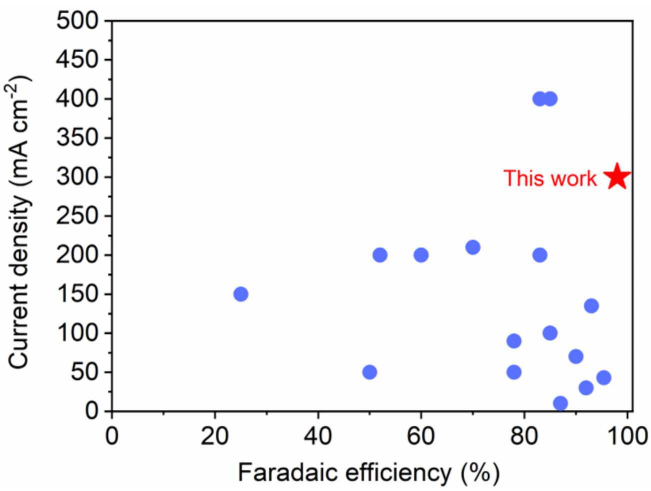

Supplementary Figure 31. The current density and Faradaic efficiency for H_2O_2 electrosynthesis by CoPc-OCNT cathode compared with high-performance catalysts recently reported (listed in Supplementary Table 7).

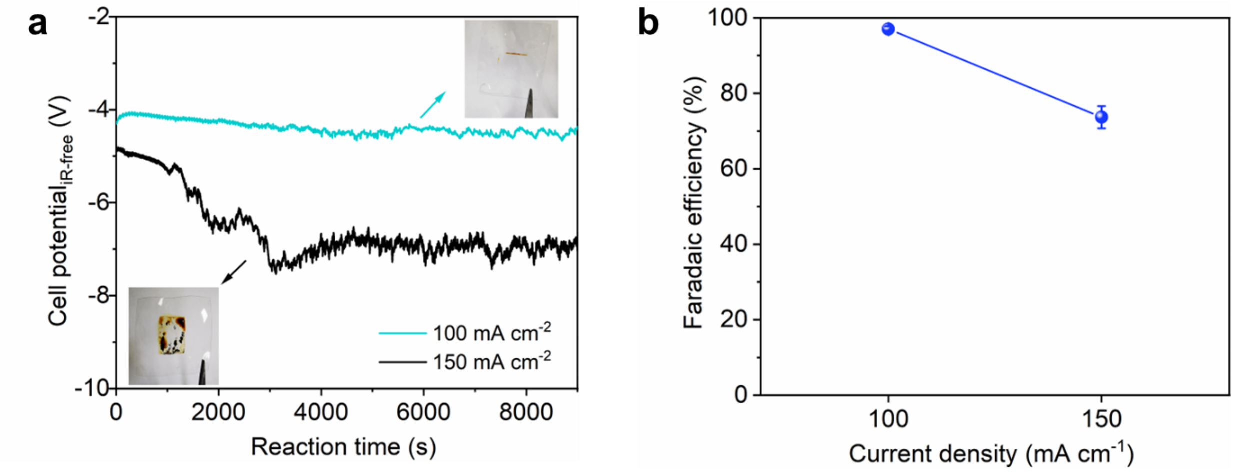

Supplementary Figure 32. The performance of H_2O_2 electrosynthesis on CuPc-OCNT cathode. a, The curves of galvanostatic electrolysis (The illustrations show the severely polluted proton exchange membrane by catalyst mixture falling from the electrode). **b,** Faradaic efficiency for producing H_2O_2 at 100 and 150 mA cm^{-2}. Experimental conditions: 1.0 M KOH (pH 13.7) was used as the catholyte with one-pass flow rate of 5 mL h^{-1}, and the anolyte of 0.5 M H_2SO_4 was circled at a rate of 33 mL h^{-1} (Data points and error bars represent the average and standard deviation of data from triplicate parallel tests, respectively).

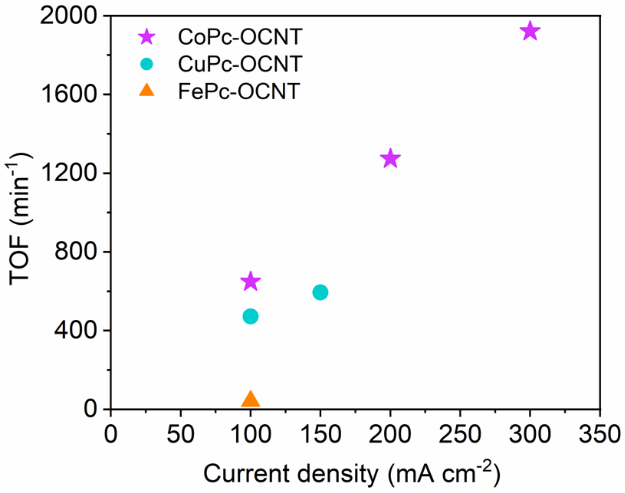

Supplementary Figure 33. Turnover frequency (TOF) values of FePc-OCNT, CoPc-OCNT and CuPc-OCNT cathodes for H_2O_2 electrosynthesis.

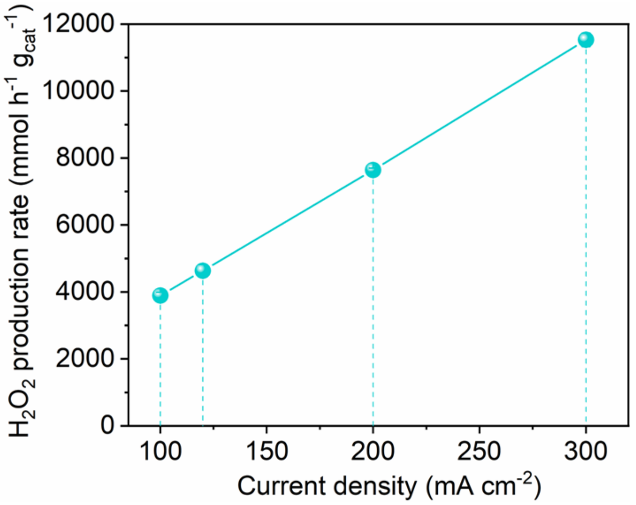

Supplementary Figure 34. H_2O_2 production rate (normalized to per mass catalyst) of CoPc-OCNT cathode at different currents density. Experimental conditions: 1.0 M KOH (pH 13.7) was used as the catholyte with one-pass flow rate of 5 mL h^{-1}, and the anolyte of 0.5 M H_2SO_4 was circled at a rate of 33 mL h^{-1} (Data points and error bars represent the average and standard deviation of data from triplicate parallel tests, respectively).

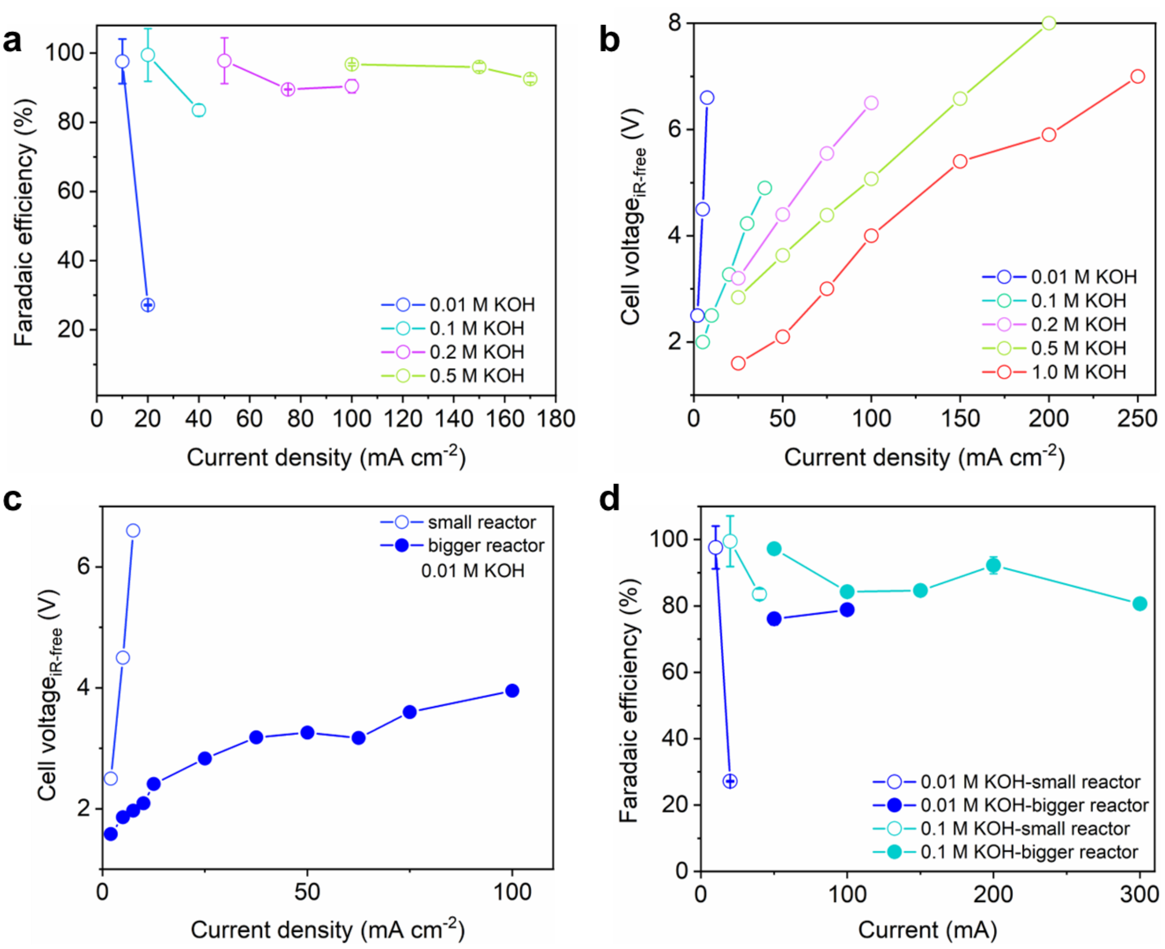

Supplementary Figure 35. The performance of H_2O_2 electrosynthesis on CoPc-OCNT catalyst using different concentrations of KOH electrolyte. a-b, Faradaic efficiency for producing H_2O_2 and cell voltages without iR compensation at different currents using 0.01, 0.1, 0.2 or 0.5 M KOH as catholyte (test conditions: small flow-cell electrolyzer with working area of 1 cm^{-2} (shown in Supplementary Fig. 28), O_2 flow rate of 20 mL min^{-1}, KOH electrolyte (0.01, 0.1, 0.2, 0.5 or 1.0 M) was used as the catholyte with one-pass flow rate of 5 mL h^{-1}, the anolyte of 0.5 M H_2SO_4 was circled at a rate of 33 mL h^{-1}). **c-d,** The comparison of cell voltages and Faradaic efficiency for producing H_2O_2 using small or bigger flow-cell electrolyzer (test conditions: bigger flow-cell electrolyzer with a working area of 4 cm^{-2} (shown in Supplementary Fig. 36), O_2 flow rate of 30 mL min^{-1}, the catholyte was one-time outflowed at a rate of 80 mL h^{-1} while the anolyte was cycled at the same flow rate, constant currents provided by a direct-current power supply). (Data points and error bars represent the average and standard deviation of data from triplicate parallel tests, respectively).

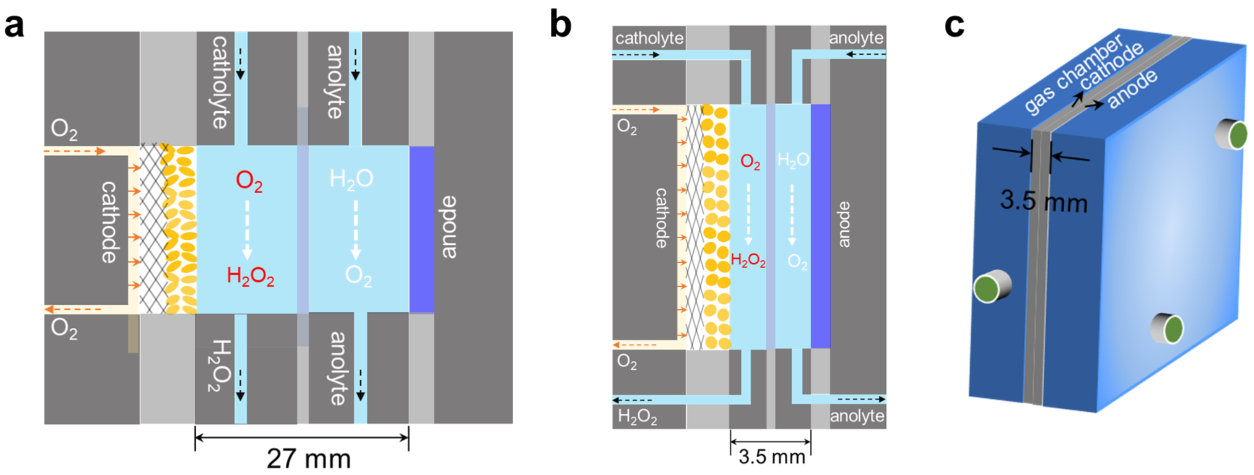

Supplementary Figure 36. The upgrading flow-cell electrolyzer for H_2O_2 electrosynthesis. a, Schematic diagram of cathodic H_2O_2 electrosynthesis and anodic water oxidation using four-chambers of the small reactor. **b,** Schematic diagram of cathodic H_2O_2 electrosynthesis and anodic water oxidation in a two-chamber of the bigger reactor. **c,** Simple drawing of bigger flow-cell electrolyzer with 3.5 mm cathode–anode distance and a 4 cm^2 working area. Test condition: the working area was 4 cm^{-2}, the catholyte was one-time outflowed at a rate of 80–100 mL h^{-1}, while the anolyte was cycled at the same flow rate. The flow rate of pure O_2 was controlled at 30 mL min^{-1}. The couples of 0.01 M KOH+0.5 M H_2SO_4, 0.1 M KOH+0.5 M H_2SO_4, 1 M KOH+0.5 M H_2SO_4, 1 M KOH+1 M KOH, 0.3 M K_2SO_4+0.3 M K_2SO_4 (pH 7.2 or 1.5 that is adjusted using H_2SO_2) were used as catholyte and anolyte to estimate the performance of H_2O_2 electrosynthesis. The constant currents were provided by a direct-current power supply.

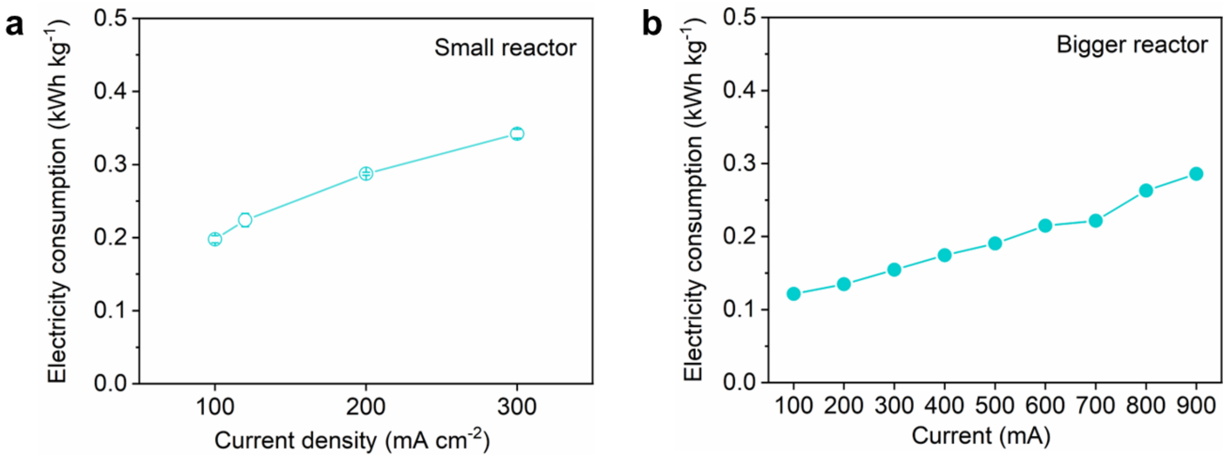

Supplementary Figure 37. Electricity consumption per kg 3 wt% H_2O_2 by H_2O_2 electrosynthesis using CoPc-OCNT cathode. a, in small reactor with 1 cm^{-2} working area, and the couple of 1 M KOH+0.5 M H_2SO_4 was used as the catholyte and anolyte at the flow rates of 5 and 33 mL min^{-1}, respectively. **b,** in bigger reactor with a 4 cm^{-2} working area, and the couple of 1 M KOH+0.5 M H_2SO_4 was used as the catholyte and anolyte at a flow rate of 100 mL min^{-1}.

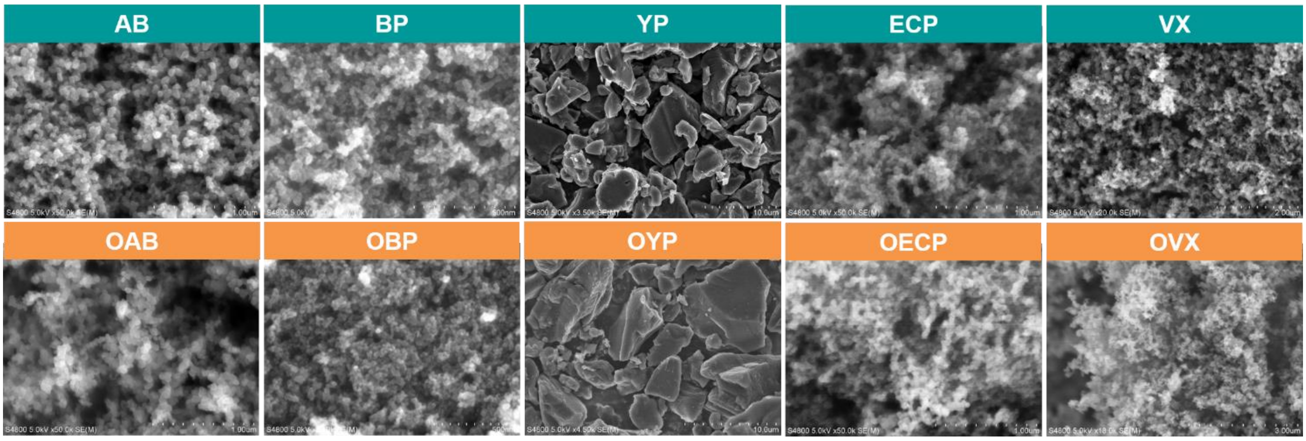

Supplementary Figure 38. Morphology characterization of five types of commercial carbon materials. SEM images of five types of commercial carbon including Timcal acetylene black (AB), Cabot black pearl 2000 (BP), Kurary YP-80F (YP), Ketjenblack ECP-600JD (ECP) and Cabot VXC72 (VX), and their counterparts after the oxidization treatment similar to OCNT.

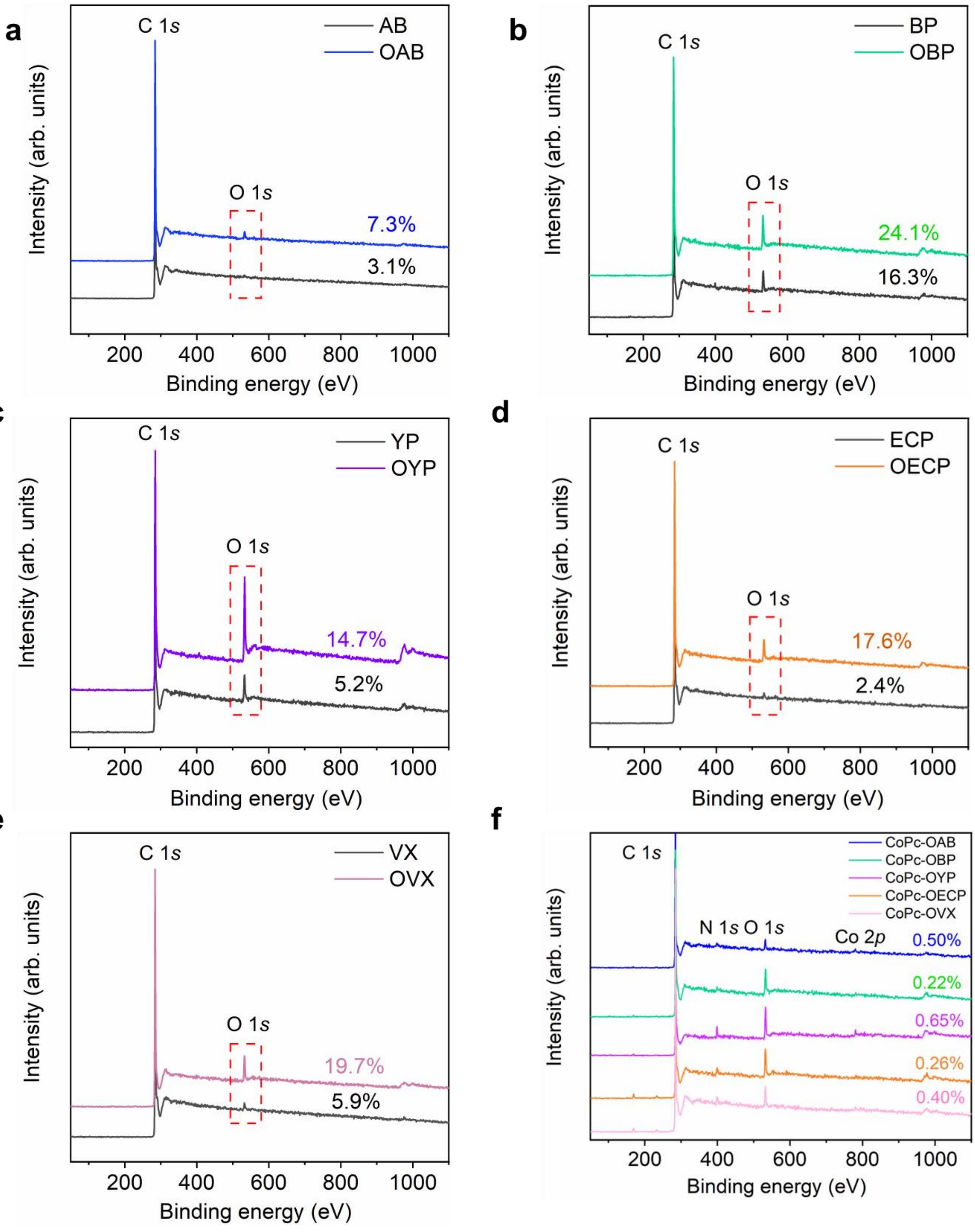

Supplementary Figure 39. Elemental constitutes of catalysts by XPS. a, AB and OAB. **b,** BP and OBP. **c,** YP and OYP. **d,** ECP and OECP. **e,** VX and OVX. **f,** CoPc supported by oxidized carbon, the numbers in figure represent the Co contents. It is worth mentioning that the XPS measurements indicate the trace concentrations of Fe, Co, Ni and Cu are detected in the five carbon and the oxidized counterparts (less 0.13% or below detection limit).

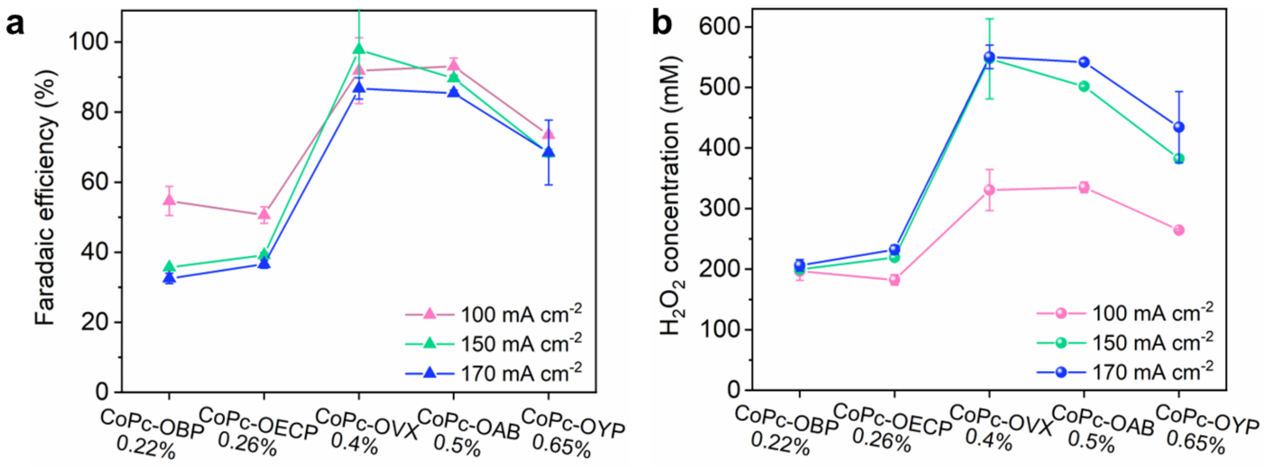

Supplementary Figure 40. The performance of H_2O_2 electrosynthesis by CoPc-OBP, CoPc-OECP, CoPc-OVX CoPc-OAB and CoPc-OYP cathodes at 100, 150 and 170 mA cm^{-2} current density. a, the concentration of H_2O_2 directly outputted from the reactor. **b,** FE for H_2O_2 electrosynthesis (Test conditions: The experiments were conducted in the small reactor with 1 cm^{-2} working area, and the couple of 1 M KOH+0.5 M H_2SO_4 was used as the catholyte and anolyte at the flow rates of 5 and 33 mL min^{-1}, respectively. Data points and error bars represent the average and standard deviation of data from triplicate parallel tests, respectively).

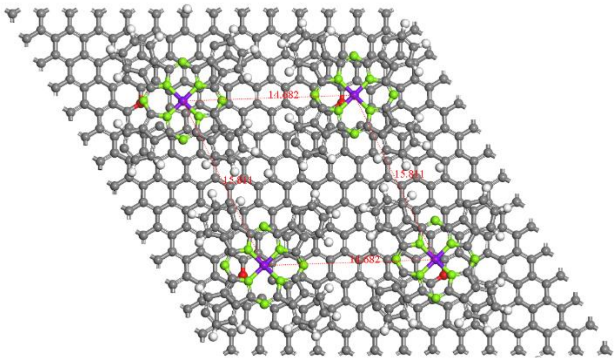

Supplementary Figure 41. The atomic structure of CoPc densely anchored on O-modified carbon support. The theoretical upper limit value is estimated to be 0.6 at% with a loading density of 0.5 molecules per nm^2, and the distance of adjacent Co sites is about 1.5–1.6 nm.

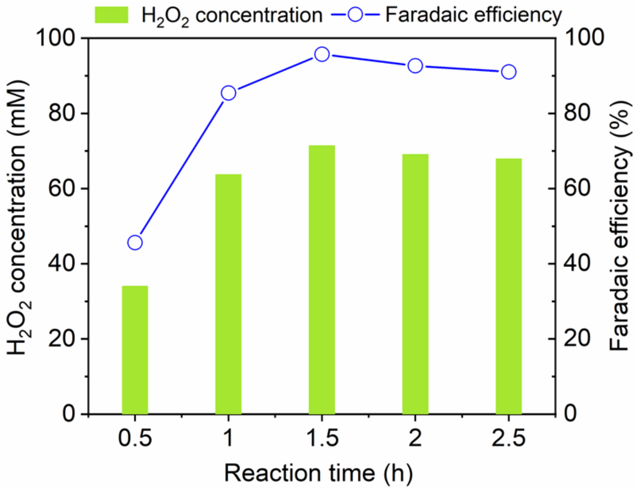

Supplementary Figure 42. The performance of H_2O_2 electrosynthesis by CoPc-OCNT cathode at 20 mA cm^{-2} using a neutral catholyte. (Test conditions: The experiments were conducted in the small reactor with 1 cm^{-2} working area, and the couple of 0.1 M K_2SO_4 catholyte (pH 7.2) +0.5 M H_2SO_4 was used as the catholyte and anolyte at the flow rates of 5 and 33 mL min^{-1}, respectively. Pure O_2 was supplied at a flow rate of 20 mL min^{-1}. Data points and error bars represent the average and standard deviation of data from triplicate parallel tests, respectively). The fact has to be pointed out that the outlet H_2O_2 product dissolved in the K_2SO_4 catholyte became acidic with pH of about 2, which could be attributed to fast H^+ transport from the anode to the cathode chamber.

Supplementary Tables

Supplementary Table 1. Atom charge variation on CoPc, CoPc/OCNT and CoPc-OCNT.

		CoPc		CoPc/OCNT		CoPc-OCNT	
	Original charge [a]	Charge [b]	ΔC [c]	Charge	ΔC [c]	Charge [b]	ΔC [c]
Co	9	8.102	-0.898	8.388	-0.612	7.798	-1.202
O(OCNT)	6	/	/	6.837	0.837	7.025	1.025
O1 (*OOH)	6	6.244	0.244	6.288	0.288	6.356	0.356
O2 (*OOH)	6	6.405	0.405	6.532	0.532	6.389	0.389
H (*OOH)	1	0.586	-0.414	0.411	-0.589	0.568	-0.432

[a] Original charge and [b] Charge: Valence electron numbers, [c] Δ*C*: Negative (in red) and positive (in blue) values represent electron loss and gain, respectively.

Supplementary Table 2. Elemental contents of CoPc-OCNT catalysts measured by X-ray photoelectron spectroscopy.

sample	C content (%)	O content (%)	N content (%)	Co content (%)
OCNT	92.00	7.01	0.99	/
CoPc-OCNT-5	90.46	7.26	2.04	0.23
CoPc-OCNT-10	89.75	7.65	2.31	0.30
CoPc-OCNT-15	90.01	7.05	2.58	0.36
CoPc-OCNT-20	88.45	7.22	3.87	0.45

/ represents below the detection limit.

Supplementary Table 3. Co loadings of CoPc-OCNT catalysts measured by inductively coupled plasma-mass spectrometry.

sample	Co content (wt%)
CoPc-OCNT-5	0.19
CoPc-OCNT-10	0.59
CoPc-OCNT-15	0.93
CoPc-OCNT-20	1.17

Supplementary Table 4. Analysis on Fourier transform infrared spectra of CoPc-OCNT.

Peaks	Assignments
435 cm^{-1}	C–C out-of-plane bending
516 cm^{-1}	C–C out-of-plane bending
575 cm^{-1}	C–C out-of-plane bending
731 cm^{-1}	C–H out-of-plane bending
756 cm^{-1}	C–H out-of-plane bending
779 cm^{-1}	C–H out-of-plane bending
873 cm^{-1}	C–H out-of-plane bending
915 cm^{-1}	Metal–N
949 cm^{-1}	C–H out-of-plane bending
1,090 cm^{-1}	C–N stretching
1,122 cm^{-1}	C–H in-plane bending
1,165 cm^{-1}	C–H in-plane bending
1,290 cm^{-1}	C–H, C–N stretching
1,332 cm^{-1}	C–H, C–N stretching
1,425 cm^{-1}	C–H, C–N stretching
1,524 cm^{-1}	C–H, C–N stretching

Supplementary Table 5. Extended X-ray absorption fine structure fitting parameters for Co K-edge of CoPc-OCNT, standard Co foil and CoPc reference (S_0^2=0.70).

Samples	Shell	N	R(Å)	σ^2	ΔE_0 (eV)	R factor
Co foil	Co–Co	12	2.49±0.01	0.0063	7.1±0.4	0.0013
CoPc	Co–N	4.6±0.7	1.90±0.1	0.0041	6.6±2.2	0.0054
	Co–N–C	3.9±2.1	3.24±0.2	0.0041		
	Co–C–N	8.7±2.6	2.94±0.2	0.0041		
CoPc-OCNT	Co–N	4.3±0.3	1.92±0.01	0.0011	5.1±1.4	0.0153
	Co–N–C	3.7±1.1	3.27±0.02	0.0019		
	Co–C–N	8.3±1.1	2.94±0.01	0.0031		

N: coordination numbers; R: bond distance; σ^2: Debye-Waller factors; ΔE_0: the inner potential correction; R factor: goodness of fit. The Co Foil is taken as standard sample to correct the absorption edge position of Co atom in tested CoPc-OCNT. Only the nearest neighbor shell is considered in the fitting of the Co foil to ensure accurate S_0^2.

Supplementary Table 6. The onset potential, overpotential and H_2O_2 selectivity (0.45–0 V vs RHE) measured in alkaline (0.01 M KOH) and neutral (0.1 M K_2SO_4) electrolytes.

	Catalysts	Onset potential (V) [a]	Overpotential (mV) [b]	H_2O_2 selectivity (%)
Alkaline	FePc-OCNT	0.814	/	75-81%
	CoPc-OCNT	0.771	/	95-100%
	CuPc-OCNT	0.751	9	86-91%
	OCNT	0.724	36	70-61%
Neutral	FePc-OCNT	0.689	11	65-71%
	CoPc-OCNT	0.597	101	85-94%
	CuPc-OCNT	0.594	106	90-93%
	OCNT	0.458	242	50-89%

[a] The onset potential is identified as the potential at –0.1 mA cm^{-2} of H_2O_2 partial current.

[b] The overpotential is identified as the potential difference with the standard equilibrium potential for two-electron ORR that is 0.76 V vs RHE in alkaline solution and 0.70 V vs RHE in neutral solution.

/ represents negligible overpotential.

Supplementary Table 7. The current density and Faradaic efficiency (FE) for H_2O_2 electrosynthesis via ORR by high-performance carbon or metal-based catalysts recently reported.

Catalysts	Current density (mA cm^{-2})	FE (%)	References
Mesoporous carbon (Meso-carbon)	150	25	6
Carbon black	135	93	7
	200	60	
Hydrophobic N-doped carbon (Hydro-NPC)	200	83	8
Boron-doped carbon (B–C)	400	85	9
Na^+/carbon black	400	83	10
Pd clusters	10	87	11
O-C(Al)	30	92	12
Fe-CNT	43	95.4	13
Co–N–C	50	50	14
$CoN_4(O)$	50	78	15
PtP_2	200	52	16
In SA/NSBC	90	78	17
$Mg_3(HITP)_2$	100	85	18
CoN_4/VG	210	70	19
$Ni-N_2O_2$	70	90	20

Supplementary Table 8. The performance comparison with high-performance carbon or metal-based catalysts recently reported for H_2O_2 electrosynthesis via ORR.

Catalysts	H_2O_2 production rate mmol g^{-1} h^{-1}	Applied current density or potentials	FE or H_2O_2 selectivity	References
rGO/PEI	106.4	0.74 V vs RHE	85.9%	21
Carbon defect	224.8	0.2 V vs RHE	>43.6%	22
Mesoporous carbon (Meso-carbon)	561.7	0.1 vs RHE	>70%	6
N-doped porous carbon (NPC)	570.1	0.2 V vs RHE	37–65%	23
Carbon black	3,565	15–200mA cm^{-2}	~90%	7
Hydrophobic N-doped carbon (Hydro-NPC)	8,530	200 mA cm^{-2}	83%	8
N-FLG	9,660	/	100%	5
Co@N-doped carbon (Co@NC)	49	0.5 V vs RHE	~98%	24
$CoN_4(O)$	418	50 mA	75–80%	15
Co–N–C	4,330	50 mA cm^{-2}	30–70%	14
Pt-CuSx	546	/	92–96%	25
Fe-CNT	1,600	43 mA cm^{-2}	95.4%	13
Pd clusters	1,701	10 mA cm^{-2}	87%	11
PtP_2	2,825	150 mA cm^{-2}	78.8%	16
In SA/NSBC	6,490	90 mA cm^{-2}	~78%	17
CoN_4/VG	4,000	210 mA cm^{-2}	70%	19
TiO_{2-x}/TiC	7,190	/	93.6%	26
**CoPc-OCNT**	**11,527**	**300 mA cm^{-2}**	**98%**	**This work**

Supplementary Table 9. The parameters of high-performance liquid chromatography for detecting different pollutants.

Targets	Mobile phase	Detection wavelength
phenol	70% methanol, 30% H_2O	271 nm
bisphenol A	70% methanol, 30% H_2O	280 nm
levofloxacin	35% acetonitrile, 45% oxalic acid and 20% methanol	271 nm
		255 nm

Note: the flow rate of mobile phase is 1.0 mL min^{-1}.

Supplementary References

1. Kulkarni, A., Siahrostami, S., Patel, A. & Norskov, J.K. Understanding catalytic activity trends in the oxygen reduction reaction. *Chem. Rev.* **118**, 2302-2312 (2018).
2. Hansen, H.A., Viswanathan, V. & Nørskov, J.K. Unifying kinetic and thermodynamic analysis of 2e- and 4e- reduction of oxygen on metal surfaces. *J. Phys. Chem. C* **118**, 6706-6718 (2014).
3. Zhao, X. & Liu, Y. Origin of selective production of hydrogen peroxide by electrochemical oxygen reduction. *J. Am. Chem. Soc.* (2021).
4. Zhao, X., Levell, Z.H., Yu, S. & Liu, Y. Atomistic understanding of two-dimensional electrocatalysts from first principles. *Chem. Rev.* **122**, 10675-10709 (2022).
5. Li, L. et al. Tailoring selectivity of electrochemical hydrogen peroxide generation by tunable pyrrolic-nitrogen-carbon. *Adv. Energy Mater.* **10**, 2000789 (2020).
6. Sun, Y. et al. Efficient electrochemical hydrogen peroxide production from molecular oxygen on nitrogen-doped mesoporous carbon catalysts. *ACS Catal.* **8**, 2844-2856 (2018).
7. Xia, C., Xia, Y., Zhu, P., Fan, L. & Wang, H. Direct electrosynthesis of pure aqueous H_2O_2 solutions up to 20% by weight using a solid electrolyte. *Science* **366**, 226-231 (2019).
8. Cao, P. et al. Durable and selective electrochemical H_2O_2 synthesis under a large current enabled by the cathode with highly hydrophobic three-phase architecture. *ACS Catal.* **11**, 13797-13808 (2021).
9. Xia, Y. et al. Highly active and selective oxygen reduction to H_2O_2 on boron-doped carbon for high production rates. *Nat. Commun.* **12**, 4225 (2021).
10. Fan, L. et al. CO_2/carbonate-mediated electrochemical water oxidation to hydrogen peroxide. *Nat. Commun.* **13**, 2668 (2022).
11. Chang, Q. et al. Promoting H_2O_2 production via 2-electron oxygen reduction by coordinating partially oxidized Pd with defect carbon. *Nat. Commun.* **11**, 1-9 (2020).
12. Yang, Q. et al. Atomically dispersed Lewis acid sites boost 2-electron oxygen reduction activity of carbon-based catalysts. *Nat. Commun.* **11**, 5478 (2020).
13. Jiang, K. et al. Highly selective oxygen reduction to hydrogen peroxide on transition metal single atom coordination. *Nat. Commun.* **10**, 3997 (2019).
14. Sun, Y. et al. Activity-selectivity trends in the electrochemical production of hydrogen peroxide over single-site metal-nitrogen-carbon catalysts. *J. Am. Chem. Soc.* **141**, 12372-12381 (2019).
15. Jung, E. et al. Atomic-level tuning of Co-N-C catalyst for high-performance electrochemical H_2O_2 production. *Nat. Mater.* **19**, 436-442 (2020).
16. Li, H. et al. Scalable neutral H_2O_2 electrosynthesis by platinum diphosphide nanocrystals by regulating oxygen reduction reaction pathways. *Nat. Commun.* **11**, 1-2 (2020).
17. Zhang, E. et al. Engineering the local atomic environments of indium single-atom catalysts for efficient electrochemical production of hydrogen peroxide. *Angew Chem. Int. Ed. Engl.* **61**, 202117347 (2022).

18. Dong, K. et al. Conductive two-dimensional magnesium metal–organic frameworks for high-efficiency O_2 electroreduction to H_2O_2. *ACS Catal.* **12**, 6092-6099 (2022).
19. Lin, Z. et al. Atomic Co decorated free-standing graphene electrode assembly for efficient hydrogen peroxide production in acid. *Energy Environ. Sci.* **15**, 1172-1182 (2022).
20. Wang, Y. et al. High-efficiency oxygen reduction to hydrogen peroxide catalyzed by nickel single-atom catalysts with tetradentate N_2O_2 coordination in a three-phase flow cell. *Angew. Chem. Int. Ed. Engl.* **59**, 13057-13062 (2020).
21. Xiao, X. et al. Enhancing the selectivity of H_2O_2 electrogeneration by steric hindrance effect. *ACS Appl. Mater. Interfaces* **10**, 42534-42541 (2018).
22. Han, L. et al. In-plane carbon lattice-defect regulating electrochemical oxygen reduction to hydrogen peroxide production over nitrogen-doped graphene. *ACS Catal.* **9**, 1283-1288 (2019).
23. Sun, Y. et al. Structure, activity, and faradaic efficiency of nitrogen-doped porous carbon catalysts for direct electrochemical hydrogen peroxide production. *ChemSusChem* **11**, 3388-3395 (2018).
24. Lenarda, A. et al. Selective electrocatalytic H_2O_2 generation by cobalt@N-doped graphitic carbon core-shell nanohybrids. *ChemSusChem* **12**, 1664-1672 (2019).
25. Shen, R. et al. High-concentration single atomic Pt sites on hollow CuS_x for selective O_2 reduction to H_2O_2 in acid solution. *Chem* **5**, 2099-2110 (2019).
26. Xu, Z. et al. Enhanced electrochemical H_2O_2 production via two-electron oxygen reduction enabled by surface-derived amorphous oxygen-deficient TiO_{2-x}. *ACS Appl. Mater. Interfaces* **13**, 33182-33187 (2021).
