# Supplementary material for: Metal single-site catalyst design for electrocatalytic production of hydrogen peroxide at industrial-relevant currents
Source: Nat Commun. 2023 Jan 12;14:172. doi: 10.1038/s41467-023-35839-z (PMC9837053; doi:10.1038/s41467-023-35839-z)
Supplement: Supplementary file 3 — Reporting Summary [file 41467_2023_35839_MOESM3_ESM.pdf]

nature portfolio

Corresponding author(s): Jingguang G. Chen, Xie Quan

Last updated by author(s): Dec 20, 2022

Reporting Summary

Nature Portfolio wishes to improve the reproducibility of the work that we publish. This form provides structure for consistency and transparency in reporting. For further information on Nature Portfolio policies, see our Editorial Policies and the Editorial Policy Checklist.

Statistics

For all statistical analyses, confirm that the following items are present in the figure legend, table legend, main text, or Methods section.

n/a	Confirmed	
☐	☒	The exact sample size (n) for each experimental group/condition, given as a discrete number and unit of measurement
☐	☒	A statement on whether measurements were taken from distinct samples or whether the same sample was measured repeatedly
☐	☒	The statistical test(s) used AND whether they are one- or two-sided<br>*Only common tests should be described solely by name; describe more complex techniques in the Methods section.*
☒	☐	A description of all covariates tested
☐	☒	A description of any assumptions or corrections, such as tests of normality and adjustment for multiple comparisons
☐	☒	A full description of the statistical parameters including central tendency (e.g. means) or other basic estimates (e.g. regression coefficient) AND variation (e.g. standard deviation) or associated estimates of uncertainty (e.g. confidence intervals)
☒	☐	For null hypothesis testing, the test statistic (e.g. F, t, r) with confidence intervals, effect sizes, degrees of freedom and P value noted<br>*Give P values as exact values whenever suitable.*
☒	☐	For Bayesian analysis, information on the choice of priors and Markov chain Monte Carlo settings
☒	☐	For hierarchical and complex designs, identification of the appropriate level for tests and full reporting of outcomes
☒	☐	Estimates of effect sizes (e.g. Cohen's d, Pearson's r), indicating how they were calculated

Our web collection on statistics for biologists contains articles on many of the points above.

Software and code

Policy information about availability of computer code

Data collection	The softwares of CHI760E, jwexp132, TEM Imaging&Analysis (TIA) Offline, Advantage, OPUS 6.5 and SmartLab were used to collect the data in this study.
Data analysis	The softwares of OriginPro 2019b, p4vasp, VESTA, Materials Studio 8.0, wxDragon, XPSPEAK41, Autosorb, Jade6, Athna and Artemis were used to analyse the data in this study.

For manuscripts utilizing custom algorithms or software that are central to the research but not yet described in published literature, software must be made available to editors and reviewers. We strongly encourage code deposition in a community repository (e.g. GitHub). See the Nature Portfolio guidelines for submitting code & software for further information.

Data

Policy information about availability of data

All manuscripts must include a data availability statement. This statement should provide the following information, where applicable:

- Accession codes, unique identifiers, or web links for publicly available datasets
- A description of any restrictions on data availability
- For clinical datasets or third party data, please ensure that the statement adheres to our policy

The data supporting the conclusions of this study are present in the paper and the Supplementary Information. The raw data sets used for the presented analysis within the current study are available from the corresponding authors upon reasonable request. Source data are provided with this paper.

Human research participants

Policy information about studies involving human research participants and Sex and Gender in Research.

Reporting on sex and gender	*Use the terms sex (biological attribute) and gender (shaped by social and cultural circumstances) carefully in order to avoid confusing both terms. Indicate if findings apply to only one sex or gender; describe whether sex and gender were considered in study design whether sex and/or gender was determined based on self-reporting or assigned and methods used. Provide in the source data disaggregated sex and gender data where this information has been collected, and consent has been obtained for sharing of individual-level data; provide overall numbers in this Reporting Summary. Please state if this information has not been collected. Report sex- and gender-based analyses where performed, justify reasons for lack of sex- and gender-based analysis.*
Population characteristics	*Describe the covariate-relevant population characteristics of the human research participants (e.g. age, genotypic information, past and current diagnosis and treatment categories). If you filled out the behavioural & social sciences study design questions and have nothing to add here, write "See above."*
Recruitment	*Describe how participants were recruited. Outline any potential self-selection bias or other biases that may be present and how these are likely to impact results.*
Ethics oversight	*Identify the organization(s) that approved the study protocol.*

Note that full information on the approval of the study protocol must also be provided in the manuscript.

Field-specific reporting

Please select the one below that is the best fit for your research. If you are not sure, read the appropriate sections before making your selection.

☐ Life sciences ☐ Behavioural & social sciences ☒ Ecological, evolutionary & environmental sciences

For a reference copy of the document with all sections, see nature.com/documents/nr-reporting-summary-flat.pdf

Ecological, evolutionary & environmental sciences study design

All studies must disclose on these points even when the disclosure is negative.

Study description	Our study calculated the thermodynamic data about catalytic reactions using a density functional method, and experimentally synthesized the solid materials for catalyzing the reactions in solid/liquid/gas interfaces for producing the liquid product.
Research sample	This is not relevant to our study, because our research focused on the synthetic catalytic materials.
Sampling strategy	No sample-size calculation was performed. We note that the experimental measurements for electrocatalytic reactions were repeated three times at a time to ensure data validation using the standard deviation method, and the measurements for each data point were also repeated three times using independent research samples.
Data collection	The data in this study were collected by analytical instruments with fixed parameters that were described in the submitted manuscript. The data were recorded by Dr. Cao using the software of OriginPro 2019b, the first author of this manuscript.
Timing and spatial scale	The data in this study were collected from Jan. 2020 to Apr. 2022. The experiments were done in one laboratory.
Data exclusions	No data were excluded from the analyses.
Reproducibility	We synthesized the main catalysts in this study five times in a time span up to two years, and confirmed the consistence of performance. All attempts to repeat the experiment are successful.
Randomization	This is not relevant to our study, because our research does not involve sample allocation.
Blinding	Blinding was not relevant to our study, because the research samples in our study were non-living synthetic materials, and the experimental data were mechanically acquisited by analytical instruments.

Did the study involve field work? ☐ Yes ☒ No

Reporting for specific materials, systems and methods

We require information from authors about some types of materials, experimental systems and methods used in many studies. Here, indicate whether each material, system or method listed is relevant to your study. If you are not sure if a list item applies to your research, read the appropriate section before selecting a response.

Materials & experimental systems

n/a	Involved in the study
☒	☐ Antibodies
☒	☐ Eukaryotic cell lines
☒	☐ Palaeontology and archaeology
☒	☐ Animals and other organisms
☒	☐ Clinical data
☒	☐ Dual use research of concern

Methods

n/a	Involved in the study
☒	☐ ChIP-seq
☒	☐ Flow cytometry
☒	☐ MRI-based neuroimaging
